# Epidemic thresholds and human mobility

**DOI:** 10.1038/s41598-023-38395-0

**Published:** 2023-07-14

**Authors:** Marta Pardo-Araujo, David García-García, David Alonso, Frederic Bartumeus

**Affiliations:** 1 Centre d’Estudis Avançats de Blanes (CEAB-CSIC), Blanes, Spain; 2 Consorcio de Investigación Biomédica en Red de Epidemiología y Salud Pública (CIBERESP), Madrid, Spain; 3 Centro Nacional de Epidemiología (CNE-ISCIII), Madrid, Spain; 4 Institució Catalana de Recerca i Estudis Avançats (ICREA), Barcelona, Spain; 5 Centre de Recerca Ecològica i Aplicacions Forestals (CREAF), Barcelona, Spain

**Keywords:** Mathematics and computing, Infectious diseases

## Abstract

A comprehensive view of disease epidemics demands a deep understanding of the complex interplay between human behaviour and infectious diseases. Here, we propose a flexible modelling framework that brings conclusions about the influence of human mobility and disease transmission on early epidemic growth, with applicability in outbreak preparedness. We use random matrix theory to compute an epidemic threshold, equivalent to the basic reproduction number $R_{0}$, for a SIR metapopulation model. The model includes both systematic and random features of human mobility. Variations in disease transmission rates, mobility modes (i.e. commuting and migration), and connectivity strengths determine the threshold value and whether or not a disease may potentially establish in the population, as well as the local incidence distribution.

## Introduction

Outbreaks of infectious diseases pose a threat to public health worldwide and may disrupt any type of human activity. Reliable and versatile tools to predict and gauge the dynamics and possible spread of a disease are thus critical for the adequate preparedness of public health systems. This is specially important during the early stages of disease outbreaks, when little information may be available on the epidemiology of the disease^1–3^.

The basic reproduction number ($R_{0}$) is probably the most widely extended indicator in health systems for assessing the risk of infectious diseases^4–6^. It serves as an epidemic threshold, valuable for assessing the severity of possible outbreaks of the disease. The $R_{0}$ is defined as the number of infections caused by a typical infected individual in a fully susceptible population. Whenever this quantity is greater than 1, the disease is expected to follow an initial exponential growth if a small number of infected individuals is introduced in the system. If it is smaller than 1, the disease is expected to die out eventually in the absence of external factors that increase its transmissibility.

As seen in the recent COVID-19 pandemic, one of the crucial factors determining the spread and control of infectious diseases is the movement of individuals. The scales^7–10^, volume^11–13^, and complexity^14–16^ of human mobility patterns bring new challenges to the understanding of the $R_{0}$, and more generally, to outbreak predictive capacity. Nonetheless, theoretical developments show how crucial epidemic information can be distilled in a simpler manner from seemingly complicated transport networks^17–20^.

We propose a modelling framework for the analysis of the possibility of spread of a disease across a metapopulation, consisting on local populations linked by a weighted network. Two kinds of human movement between these are contemplated in the model, in search of an accurate understanding on how different types and scales of mobility may affect the $R_{0}$ of the disease. To model the variability inherent to spatial processes and human activities, we assume that the strengths of the connections between the local populations are randomly distributed. This allows us to focus on general properties of the model, rather than in specific instances of it. Using tools from random matrix theory (RMT^21–23^), we are able to obtain explicit predictions on the possibility of spread of the disease in terms of only a few parameters of the underlying distributions. In particular, we find that even though the systems are formulated in terms of random quantities, the predicted epidemic threshold of the disease only depends on deterministic indicators.

We consider a SIR epidemic model that consists of *N* coupled local populations, or patches. Three different processes may modify the number of infected individuals at each patch *i*: (a) the natural dynamics of the disease, consisting of contagions of susceptible from infected individuals from the patch (at a rate $\beta _{i}$ of transmission of the disease) and recovery of infected individuals (at a rate $\gamma _{i}$, that combines the rate of true recovery from the disease and the natural death rate of the population), (b) contagions of susceptibles from infected individuals from other patches *j* (at a rate $\beta _{j}c_{ji}$, that factors the local disease transmission rate $\beta _{j}$ and a corrective term $c_{ji}$), and (c) arrivals/departures of infected individuals from/to other patches *j* (at rates $m_{ij}$ and $m_{ji}$ respectively). These assumptions are captured in the following set of differential equations

1 $$\begin{aligned} \frac{dI_i}{dt} = \beta _i \frac{S_i}{N_i}I_{i} - \gamma _iI_i + \sum _{j=1}^{N} \beta _{j}c_{ji}\frac{S_{i}}{N_{i}}I_{j} - \sum _{j=1}^N m_{ji} I_i + \sum _{j=1}^N m_{ij} I_j, \qquad i\in \{1,\dots ,N\}, \end{aligned}$$

where $S_{i}$ and $N_{i}$ denote the susceptible and total populations at each of the *N* nodes of the network. We assume that the total population of the system remains constant over time; see Supplementary Sect. 1 for more details and the full system of equations.

The interaction terms $c_{ij}$ may be associated to short-term mobility based on back-and-forth displacements between the patches of the system, while the movement rates $m_{ij}$ represent displacements from one patch to another with no returns, suggesting temporally delayed or long-term mobility compared to back-and-forth motion. We will refer to these types of mobility as *commuting* and *migration* respectively, and refer to them as the *connectivity* network as a whole.

The two mobility related terms in Eq. (1) are standard in models of infectious diseases^24,25^ and are usually analyzed separately. The force of infection generated by back-and-forth interactions (factored here as the product of the local term $\beta _{j}$ and the corrective factor $c_{ji}$, quantifying the force of infection on patch *i* generated by interactions with individuals from patch *j*) was introduced by Lajmanovich and Yorke^26^ and has been studied by Lloyd and May, among several authors^27–31^, and migratory rates among populations have been treated extensively before^32–35^. Locally, the disease evolves at each node according to the classical SIR model^4,36,37^. The two types of mobility considered here (comprising “returners” and “explorers”) have also been observed in real-world mobility networks, and are expected to have different effects in spreading processes^9^.

While the epidemic spread in random networks has been subject of intensive investigations^38–41^, the novelty in our approach is the random nature of the parameters describing the connectivity between nodes. We draw the commuting and migration coefficients $c_{ij}$ and $m_{ij}$ from two arbitrary probability distributions, modelling randomness in the strength of the connections rather than on their occurrence (although, in particular, the absence of a connection is modelled by a zero-strength connection). This accounts for random connectivity and spatial heterogeneity, and allows for an innovative application of RMT techniques, as we demonstrate below. These techniques have been successfully employed in the study of ecological communities by Allesina et al.^42–44^ and others^45–48^, generalizing in various directions the famous stability-complexity criterion of May^49^.

We follow the approach developed in Refs.^50,51^ and study an equivalent indicator to the $R_{0}$ by means of the Jacobian matrix *J* of the linearized *infected subsystem* of the model (1) around the disease-free equilibrium (DFE). More precisely, we are interested in computing the largest real part of the eigenvalues of *J*, denoted by *s*(*J*), that determines the initial behaviour of the disease:

$$\begin{aligned} {\left\{ \begin{array}{ll} \,s(J)>0: &{} \quad \text {the disease may spread over the system (instability),} \\ \,s(J)<0: &{} \quad \text {the disease will die out eventually (stability).} \end{array}\right. } \end{aligned}$$

Note that *s*(*J*) does not need to match the epidemiological definition of $R_{0}$, in the sense that it does not measure the average number of secondary infections generated by a primary one^50^; it does however provide an equivalent epidemic threshold for disease spread. Another common tool in the study of the stability of the model is the next-generation matrix of the system^51^; we show in Supplementary Sect. 1.2 how our methods could be used equivalently in this context.

We also analyze the infectious disease dynamics described by Eq. (1) by making several more general assumptions. We study each of these generalizations using RMT, both analytically and numerically. For instance, we incorporate randomness in the epidemiological parameters and we target specific modifications of the connectivity network, which could reflect several epidemic scenarios and public health measures (e.g. increased transmission at particular locations, heterogeneous vaccination landscapes, mobility restrictions). In general, we find that qualitatively different behaviours may result from these variations, providing valuable guidance for public health decision-making.

## Results

### Epidemic threshold

For simplicity, we first compute the epidemic threshold for a system with the same transmission and recovery rates ($\beta$ and $\gamma$ respectively) across the *N* nodes of the system. In the subsequent sections we lift this restriction and analyze more general situations.

**Figure 1:**
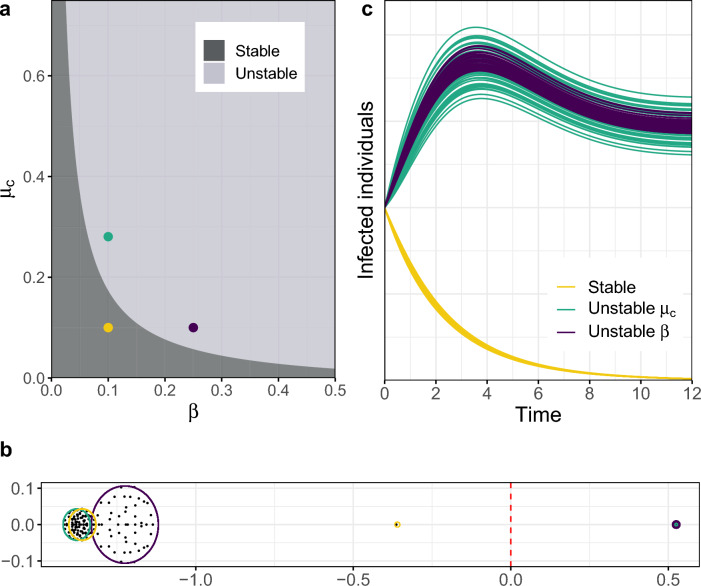
Epidemic spread induced by disease transmission and human connectivity. The epidemic threshold Eq. (2) depends on the transmission and recovery rate of the disease, as well as the average commuting flow of the network. We show examples, for a system with $N=50$ and $\gamma = 0.95$, of three different scenarios in terms of these parameters: a stable scenario where the outbreak dies out (yellow), an epidemic scenario caused by the transmission of the disease (purple), an epidemic scenario for a disease with low transmission, caused by the high connectivity between the nodes of the network (green). (**a**) Phase diagram of the stability of the system in terms of the transmission rate $\beta$ and the average commuting flow $\mu _{c}$. (**b**) Eigenvalue distribution of the Jacobian matrix of the infected subsystem Eq. (1) for the three scenarios. The corresponding epidemic thresholds are given by the three outlier eigenvalues lying on the right of the circle distributions; the outlier of the stable system has negative real part and those of the unstable scenarios have positive real parts. The bulk distribution of the increased transmission scenario is modified due to the resulting increase in the variance of the network (see Eq. (8)). (**c**) Evolution of the number of infected individuals over time across the patches of the network. Both unstable systems show the same qualitative behaviour, as their epidemic thresholds coincide, and the one with higher commuting displays higher variability of infected individuals across the nodes of the network.

As explained in the introduction, we assume that the mobility coefficients $c_{ij}$ and $m_{ij}$ are drawn from two arbitrary probability distributions with mean $\mu _c$ and $\mu _m$ and variance $\sigma _c^2$ and $\sigma _m^2$, respectively. As a consequence, the Jacobian matrix of the system at DFE has randomly distributed entries (see Eq. (6)). The asymptotic eigenvalue distribution of this matrix can then be described using the low-rank perturbation theorem^52,53^, a generalization of the circular law of RMT^54^. As $N\rightarrow \infty$, most of the eigenvalues (the bulk) almost surely distribute uniformly on a circle of radius centered at $\beta \mu _c - \mu _m N + \beta - \gamma$ with radius $\sigma \sqrt{N}$, where $\sigma$ encodes the joint variability of the two families of mobility coefficients (see Eq. (8)), with the exception of a single outlier eigenvalue located on the real axis at $\beta \mu _{c}(N-1) + \beta - \gamma$ (Fig. 1b, see “Methods” section for more details). Provided that the mobility coefficients are positively distributed, we find that the bulk of the eigenvalues is located on the left of the outlier (see Supplementary Sect. 1.1). Since the stability of the system is given by the real part of the rightmost eigenvalue of the system, this implies that the threshold for the possibility of spread of the disease is given by

2 $$\begin{aligned} s(J) = \beta \mu _{c}(N-1) + \beta - \gamma . \end{aligned}$$

Although only exact in the large-*N* limit, Eq. (2) shows a high degree of accuracy even for relatively small networks (typically above $N=20$ nodes, see Supplementary Sect. 1.1). Note that the particular distribution followed by the commuting and migration coefficients is irrelevant for the large-scale dynamics of the disease, as only their moments up to second order are relevant, such as their means and joint variance. This is a consequence of the well-known universality phenomenon in RMT^54^, that reduces the apparent complexity of the problem to the simple expression Eq. (2), involving only the epidemiological parameters of the system and the average commuting flow. See Fig. 1 for an example of stable and unstable systems as determined by the epidemic threshold Eq. (2).

The importance of each parameter on the spread of the disease can be interpreted from Eq. (2). If the transmission rate is greater than the depletion rate ($\beta > \gamma$), the DFE is unstable ($s(J) > 0$), resulting in a scenario of initial epidemic growth. However, if $\beta < \gamma$, the average commuting flow is a key factor for the stability of the system (Fig. 1a). The greater the difference $\gamma - \beta$ is, the easier it is to achieve stability. The rate of recovery of infected individuals $\gamma$ combines the rate of true recovery and the natural mortality rate; the relative contribution of each of these to the recovery rate influences the long-term dynamics of the disease (see Supplementary Fig. 2). Let us also note that, besides controlling the stability of the system, the size of the rightmost eigenvalue of the Jacobian *s*(*J*) also shapes the magnitude of the epidemic in a nonlinear fashion (see Supplementary Fig. 3).

Remarkably, the configuration of the migration flows does not influence the stability of the system Eq. (2). However, larger volumes of migrating individuals do result in a more variable evolution of the disease across patches (Supplementary Fig. 10). This is a consequence of the randomness of the migration coefficients: a larger average $\mu _{m}$ leaves more space for fluctuations around the mean, which results in nodes accumulating both larger and smaller proportions of incoming infected individuals. Thus, even though the overall number remains essentially the same, more migration leads to larger local heterogeneity, with locations under higher and lower epidemic stress. The combined variance of the commuting and migration flows has a similar effect: more variability in mobility translates into a more heterogeneous epidemic evolution across the nodes of the network (Supplementary Fig. 10). This is particularly relevant for the containment of the disease, as the distribution of the epidemic stress is of capital importance when facing severe outbreaks^55–57^.

### Variations in epidemiological parameters

The epidemic threshold given by Eq. (2) was obtained under the assumption that the rate of disease transmission did not change across nodes. We now relax this constraint and explore how perturbations in these parameters affect the spread of the disease across the system. These modifications could be understood as local heterogeneities in the probability of transmission produced by vaccination policies, new disease strains, non-pharmaceutical interventions or socioeconomic inequalities, for instance.

**Figure 2:**
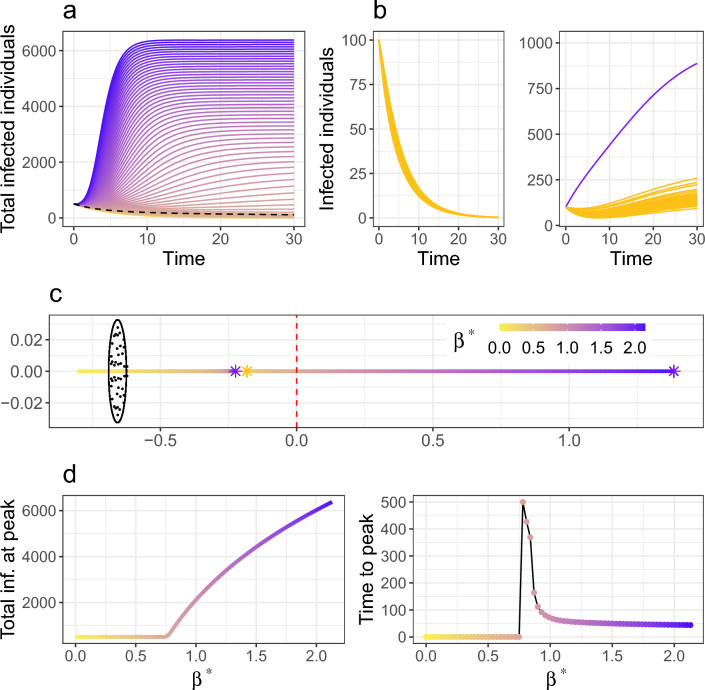
Epidemic spread due to a variation of disease transmission in one patch. Modifying the transmission rate at one patch changes the epidemic threshold Eq. (2) to the more complicated expression Eq. (9). Transmission changes from $\beta$ to $\beta ^{*}$, ranging from no transmission at the perturbed patch (yellow) to an increased transmission (purple). Disease transmission occurs at a rate $\beta =0.15$ and the network has $N=50$ patches. (**a**) Sum of infected individuals across all patches over time for different values of $\beta ^{\star }$. A change of regime is represented by the dashed line defined by the threshold value of $\beta ^{\star }$. (**b**) Infected individuals over time for a system with equal transmission rates (left) and a system with an increased transmission rate at one node (right, perturbed node in purple). Even though the perturbation in transmission only affects one patch the system switches from stable to unstable. (**c**) Eigenvalue distribution for the system with different transmission rate in one patch. As the perturbation increases the epidemic threshold given by the rightmost eigenvalue increases. The stars correspond to the examples presented in (**b**), yellow for the left plot and purple for the right one. One more outlier arises for the perturbed case due to the specific form of the structure matrix (see “Methods” section). (**d**) Maximum number of infected individuals at one patch (left) and time to reach the epidemic peak (right) in terms of $\beta ^*$. The maximum increases nonlinearly as the perturbation in transmission increases, while the time to reach this maximum decreases since the initial growth is steeper for higher $\beta ^*$.

#### Local variations in transmission rates may cause global epidemic spread

Let us assume that the disease transmission rate changes from $\beta$ to $\beta ^*$ at a small subset of patches in the system. This new rate could range from no transmission (vaccination, quarantine) to an exceptionally high one (highly contagious strains, massive gatherings). Using the low-rank perturbation theorem^52,53^, we see that the bulk of the eigenvalues of the Jacobian remains the same but the location of the outlier is modified (Fig. 2c), and additional outliers are generated for every perturbed node (see Eq. (9) for an analytical expression and Supplementary Sect. 2 for more details). As expected, we find that disease spread is facilitated (obstructed) if the perturbed transmission rate $\beta ^{*}$ is lower (higher) than $\beta$ (Fig. 2a), larger perturbations resulting in higher and earlier epidemic peaks (Fig. 2d). This perturbation influences the whole system and not exclusively the node with higher transmission (Fig. 2b). Interestingly, the resulting epidemic threshold (Eq. (9)) now depends also on the mean migration flow $\mu _{m}$, although a minor effect is observed (see Supplementary Fig. 8). This contrasts with the unperturbed case Eq. (2) in which the stability of the system only depends on the average commuting rate $\mu _{c}$. Importantly, an increase in transmission at a single patch has a more drastic impact on the stability of the system than an equivalent rise distributed over several patches (see Supplementary Fig. 8).

#### Mobility (and not transmission) structures the spread of the disease

**Figure 3:**
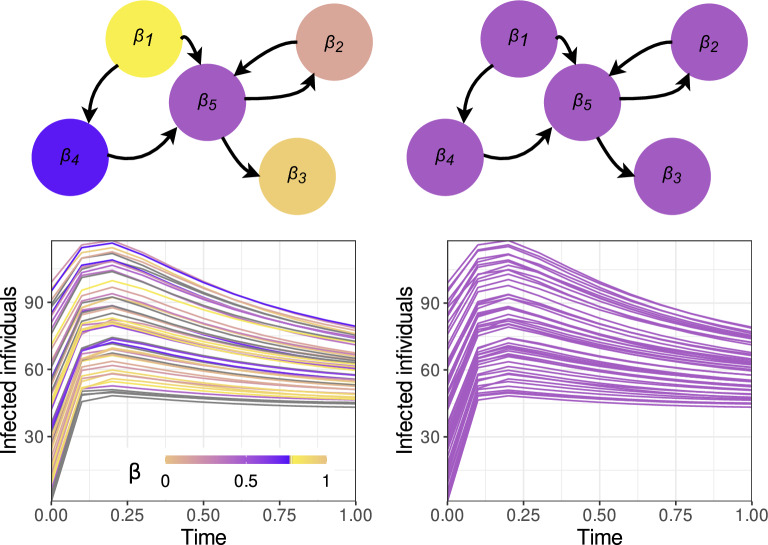
Transmission does not necessarily shape the distribution of infected individuals. Two systems, one with random transmission (left), where the transmission rate at each node is drawn from a probability distribution, and one with the same transmission at all patches (right), equal to the mean of this distribution. The network has $N=50$ nodes and the $\beta _{i}$ are drawn from a random distribution of mean $\mu _{\beta } = 0.6$ and standard deviation $\sigma _{\beta } = 0.6$. We present a schematic representation for the network (top) and the evolution of the number of infected individuals at the patches over time (bottom), colored according to the local transmission rate. Even though the transmission landscape is very different for both examples the distribution of infected individuals over the network is quite similar. In particular, the case with random transmission shows a network with zero transmission in some nodes (in yellow) in which there is an initial increase of infected individuals.

Once contagions take place, infected individuals may be redistributed through mobility flows across the network. In particular, it turns out that even fairly variable transmission landscapes result in similar disease dynamics as homogeneous ones. Figure 3 shows an instance of two systems displaying the same qualitative behaviour in terms of disease spread, caused by two contrasting transmission landscapes: one for which disease transmission rates change across the nodes of the network, and one for which the transmission rates are the same at all nodes (and equal to the average rate of the first system). In the first case, there are nodes with relatively high transmission rates (representing massive gatherings, absence of public health interventions) and nodes with no infection at all (representing for instance effective vaccination). Nevertheless, more variability in the transmission rates (either in variance or in coefficient of variation) does require a larger-sized network to achieve an accurate match to the asymptotic expression in Eq. (2) (see Supplementary Fig. 9).

The results above show that for contexts verifying our hypothesis (large networks with variable mobility flows), the mobility network is relevant in determining the distribution of infected individuals across the system. This has important consequences for disease control. For example, regions with a higher incidence of the disease may be masking the true sources of contagion, where preventive measures would be most effective. Also, extreme events or changes in policies at selected locations, that would cause localized outbreaks, may evolve into an overall unstable scenario due to human movement.

An analogous result holds whenever the rates of recovery of infected individuals $\gamma _{i}$ change across nodes. Indeed, as a consequence of the low-rank perturbation theorem^53^, we find that the location of the rightmost eigenvalue of the Jacobian matrix *J* is independent of the variance of its diagonal elements. In particular, this means that only the average recovery rate is relevant for the epidemic threshold (and not the particular distribution followed by $\gamma _{i}$, see Supplementary Sect. 2).

### Variations in mobility parameters

In this section, we extend the results derived from Eq. (2) to more general random matrix configurations. In particular, we analyze disease stability against different perturbations of the mobility flows and network arrangements.

#### Reducing commuting flows is an effective strategy for epidemic control

We first analyze how changes in the commuting flows could affect the possibility of disease spread over the network. More precisely, we remove the force of infection generated by the interactions between infected and susceptible individuals from different patches, as lockdowns have a direct effect on short-term, commuting patterns, and would be a typical control strategy for possible outbreaks. In the following, we will refer as incoming flows at patch *i* to the interactions between susceptible individuals from that patch and infected individuals from other patches, resulting in infections at patch *i*. Removing these flows at a patch thus amounts to removing the force of infection generated on it by the rest of the patches. On the other hand, we will refer as outgoing flows from patch *i* to infections caused by infected individuals from patch *i* in susceptibles residing in other patches. Restricting these flows then affects the force of infection generated by the patch on the rest of the system.

**Figure 4:**
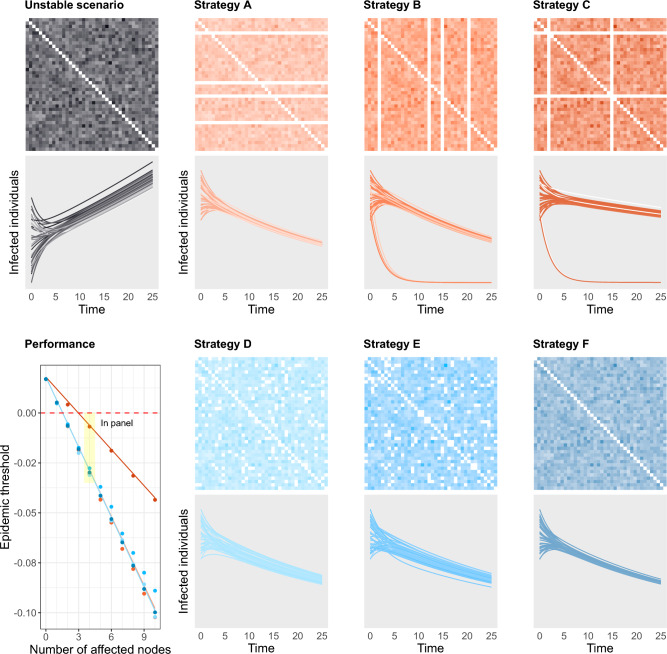
Restrictions in commuting and their impact on disease spread. We consider a base network of size $N=40$ and average migration rate $\mu _{c}=0.12$, a scenario of uncontrolled disease spread (grey). We test the six mobility restriction strategies described in this section, which all result in successfully controlling the expansion of the disease by perturbing 10% of the commuting flows of the network to $\mu _{c}^{*} = 0$. For each scenario, the top graph displays the strength of the commuting flow from patch *i* to patch *j* in its (*i*, *j*)-th cell. The brightness of the color represents the strength of the interaction, with white representing absence of interaction. Each line in the bottom graph shows the evolution of the infected population at each patch, colored according to its average incoming and outgoing commuting flow. The three targeted strategies (in shades of orange) consist of A: restricting all outgoing flows at 4 nodes, B: restricting all incoming flows at the same set of nodes as in A, C: restricting both incoming and outgoing flows at half of the nodes selected in the previous scenarios. The three random strategies (in shades of blue) consist of D: restricting randomly chosen unidirectional flows, E: restricting half as many randomly chosen flows in both directions, F: uniformly decreasing all the flows in the network. The bottom left graph shows the resulting epidemic threshold in terms of the number of perturbed nodes, as given by the analytical expressions provided by Eqs. (10)–(12) (continuous lines) and empirical computations from synthetic networks (dots).

We tested six different strategies for the containment of the disease, comprising three targeted and three non-structured restrictions (Fig. 4). These scenarios represent perturbations aimed to curb an unstable setting. We perturbed (reduced to zero) a fixed number of interactions between nodes, which gives rise to the different scenarios. The three targeted strategies consist of shutting down at selected locations A: all outgoing flows, B: all incoming flows, and C: both incoming and outgoing flows (at half of the locations). The three non-structured strategies consist of D: shutting down as many randomly chosen unidirectional interactions between patches as for the targeted scenarios (that is, we keep the flow from patch *i* to patch *j* while shutting down the flow from *j* to *i* or vice-versa), E: shutting down randomly chosen interactions between patches in both directions, and F: uniformly decreasing the strength of the interactions on the whole network. Based on our model, we obtain analytical expressions for the epidemic thresholds for all these scenarios (see Methods), which can be regarded as generalizations of Eq. (2). We also compare the effectiveness of the strategies in reducing the epidemic threshold in terms of the number of modified flows (see Fig. 4).

The three targeted strategies (A,B,C) result in considerably different local distributions of infected individuals. Those nodes for which all incoming flows have been removed show a direct decrease in the number of infected individuals (B,C); while restricting only outgoing flows (A) does not create quantitative differences between the patches (Fig. 4). Nevertheless, the initial growth of infected individuals over the system as a whole is the same for strategies A and B. This suggests that similar resources allocated to contain a disease (number of flows reduced to zero) may be equally effective (same *s*(*J*)), but result in significantly different local impact. Strategy C, combining incoming and outgoing restrictions, causes a smaller reduction in the epidemic threshold *s*(*J*) compared to strategies A and B. As the rightmost eigenvalue controls the magnitude of decrease or increase in the number of infected individuals, the velocity of decay in C is thus lower compared to A and B (Fig. 4). This indicates that it may be more effective to distribute control efforts than to concentrate them at fewer locations. As shown in Eqs. (10) and (11), the average migration flow influences the epidemic threshold for the targeted scenarios (contrary to non-perturbed scenarios), causing more significant variations for Strategy C (see Supplementary Fig. 12).

The three non-structured strategies (D,E,F) cause the exact same reduction in the epidemic threshold *s*(*J*), due to the universality of eigenvalue distributions of random matrices, even though the commuting networks are quite different (Fig. 4). Indeed, the threshold Eq. (2) depends only on the average commuting rate, and this is subject to the same variation under the three strategies. These expressions are qualitatively different from those resulting from targeted perturbations (see “Methods” section), in agreement with previous results^58^. As a remark, strategies D and E produce a higher variation between nodes than F, and larger systems are required to achieve accuracy between empirically computed thresholds and their analytical predictions (Fig. 4). In general, we find that more spread interventions cause a higher decrease in the epidemic threshold (see Fig. (4)).

#### Changes in migration shape the local incidence of the disease

As seen in Eq. (2), migratory flows do not modify the possibility of spread of the disease in the unperturbed scenario. Nevertheless, their particular configuration may influence the quantitative behaviour of the epidemic and the local distribution of infected individuals across nodes. A reason for this is that the migration flows determine the sizes of the populations at the equilibria of the system, as shown in Eq. (4).

We investigate this by testing analogous strategies to those shown in Fig. 4 for targeted and random increases in migratory flows (see Supplementary Sect. 3.2). Increased migratory flows may reflect the effect of seasonal movement patterns or crisis scenarios, hence, an increase of the so-called exploratory behaviour^9^. We find that an increment in the incoming (outgoing) migration flows at specific locations results in a larger (smaller) number of infected individuals at these nodes, respectively. A node having both incoming and outgoing large flows will display the same qualitative behaviour as the rest of the nodes in the system, as these compensate each other. Among the random strategies, those resulting in a network with higher variability (such as strategies D and E) cause a more heterogeneous epidemic evolution across nodes. In any case, the potential for disease spread and the approximate number of infected individuals in the system remain the same for all scenarios.

#### Correlated mobility flows can cause or prevent epidemic spread

The epidemic threshold Eq. (2) shows that the possibility of spread of the disease is independent of the statistical variability of the mobility networks. However, more subtle configurations of these flows can change the properties of the system. For instance, it is well-known that correlations between diagonally opposite elements in random matrices (that is, mobility flows on the same edges going in opposite directions) deform the bulk eigenvalue distributions in Fig. 1, changing the circular law to a now elliptic law^53^. However, as discussed above, only the outlier eigenvalue is responsible for the stability of the system, and thus these correlations alone are not enough to modify the epidemic threshold.

**Figure 5:**
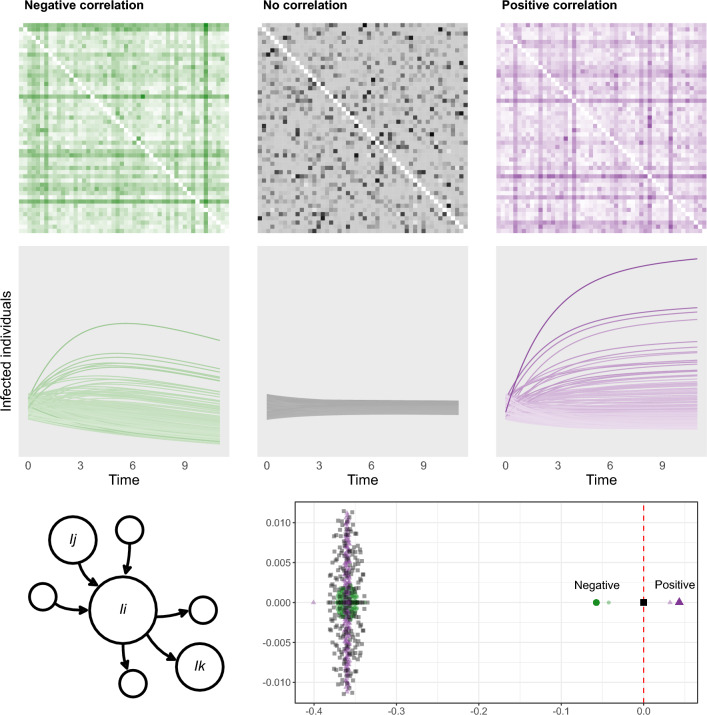
Correlated flows may determine the growth of the outbreak. Three systems with identical epidemiological parameters and underlying networks (of size $N=200$) with identical mean and variance are depicted in the same way as described in Fig. 4. The base scenario (grey) displays no correlation between incoming and outgoing flows at its nodes ($\Gamma =0$, see Eq. (14)) and shows a negligible variation over time in terms of disease spread. Setting this correlation to a negative value causes the disease to die out in the long term ($\Gamma = -\, 0.3$, green), while a positive correlation results in a scenario of epidemic growth ($\Gamma = 0.3$, purple). The effect of this correlation on the mobility flows can be identified from the networks: positively correlated networks favour the existence of nodes with both high incoming and high outgoing flows (darker horizontal and vertical lines tend to intersect at the diagonal), while in negatively correlated networks nodes with high incoming flows generally do not have high outgoing flows and vice-versa (darker horizontal and vertical lines do not intersect at the diagonal). Bottom plot shows the eigenvalues of the Jacobian matrix of the three systems shown in the scenarios (smaller, faded symbols), together with their predicted outliers as given by Eq. (15) (larger, solid symbols).

The mobility network, however, may present more complicated correlations. As observed in Ref.^59^, correlations between elements of random matrices that share a common index may modify the location of the outlier eigenvalue as well, even causing epidemic spread in systems that would have otherwise been predicted as stable. Figure 5 shows two examples of this phenomenon; more details and examples of correlated mobility networks are shown in Methods and Supplementary Sect. 3.3. It turns out that only the correlation between the incoming and outgoing connectivity at the nodes influences the potential for disease spread; its sign determines whether the mobility network fosters or prevents disease spread. The other three possible types of correlation considered in Ref.^59^ (those involving flows going in opposite directions and those involving either incoming or outgoing flows at the nodes of the network, see Supplementary Fig. 14) do not affect the stability of the system. In particular, whenever the incoming and outgoing flows at each node are positively correlated (favoring the existence of high-traffic and low-traffic nodes rather than a less coordinated arrangement), the disease spread is facilitated. Instead, mobility networks in which only the existence of *source* or *sink* nodes is favored (high correlations for only outgoing or incoming flows) display the same qualitative behaviour as uncorrelated ones. Importantly, the epidemic threshold Eq. (15) of networks with correlated mobility flows depends on the joint variance of the mobility network $\sigma$ and the average migration rate $\mu _{m}$, with higher values of these parameters leading to higher potential of disease spread.

## Discussion

For the first time, we use random matrix theory for the study of epidemic models. This novel approach allows us to compute epidemic thresholds analytically and understand the expected early epidemic growth of infectious diseases conditioned to random transmission and human mobility. Our framework addresses several known challenges for network and metapopulation infectious disease models^60–63^, and shows enough flexibility to deal with the temporal and spatially structured randomness inherent to human connectivity networks. Recent developments in RMT^59,64,65^ are yet to be exploited in this context, and may better adjust these analytical tools to more realistic scenarios. We expect epidemic models to benefit from RMT as community ecology has already done^42,43,47^.

Here, we have considered a SIR metapopulation model in a fully connected network with randomly distributed connection strengths between patches or nodes. The model includes two types of mobility among populations: commuting (individuals going back and forth between two patches) and migration (individuals moving from one patch to another). We compute an epidemic threshold, equivalent to the basic reproduction number $R_{0}$, that determines whether the establishment of an infectious disease upon an outbreak will be possible or not. This generalizes a previous expression of Lloyd and May^29^ derived for a simpler model without migration and a uniform force of infection across patches. Our result is fully explicit and, as such, allows us to identify the role of each feature of the connectivity network in the early growth of the disease. In non-perturbed random networks, we found that only the commuting flows influence the stability of the system, and may cause the spread even of diseases that would have otherwise gone extinct. Migration flows, on the other hand, shape the spatial distribution of infected individuals over the network.

More complex dependencies were observed when exploring plausible perturbations of the system. We found that the stability of systems exposed to localized variations in transmission and connectivity was influenced by the average migratory rate, and that higher variability in the mobility flows favoured the spread of the disease in correlated networks. This contrasts with the unperturbed case, where early epidemic growth was independent of these features. In general, variations leading to higher local concentrations of force of infection cause more instability than equivalent, widely disseminated distributions. This phenomenon was observed: (i) when increasing the transmission of the disease at selected locations, (ii) when evaluating different targeted and random strategies for mobility reduction, and (iii) when allowing for general correlations on the connectivity flows. This may be due to a combination of the exponential growth of infectious diseases during early stages of outbreaks and the statistical nature of the underlying network. Epidemics are expected to follow an initial exponential growth and thus a steeper rise in contagions is caused by accumulations of infected individuals. Therefore, configurations that produce larger statistical fluctuations in the network result in higher local concentrations of infected individuals, while more evenly spread distributions generate smaller, diluted increases.

Even though we can infer some generalities from our model, any sort of universal recipe for stability should be handled with caution, as the effects of the connectivity between patches are subtle and have implications on other features of the epidemic evolution. For instance, equally effective strategies to control the disease globally can produce significant differences in the magnitude of local outbreaks. Another example is observed when considering specific correlations in the mobility flows. Even if the gross number of moving individuals remains the same, particular configurations of the network may change the stability of the system. Remarkably, our results resemble previous findings in agent-based random networks^17,40,66^, in the fact that only moments up to the second order of the connectivity distributions are relevant for the epidemic thresholds.

Understanding the structure of human connectivity networks may provide useful information for the containment of outbreaks. We have seen how mobility is crucial in determining the distribution of infected individuals across the network, in addition to the natural dynamics of the disease. More precisely, we found that the incidence of the disease follows patterns associated to the connectivity of the system, that may not coincide with the spatial configuration of the transmission. For instance, geographically close locations with similar transmission rates could display contrasting disease incidence. This phenomenon is crucial to identify true sources of disease spread, and has been observed before in other transport networks^11,15,19,20^. This could be particularly useful for the design of effective control measures during the early stages of an outbreak, when the epidemiology of the disease may not be yet understood. Many of these policies are based on mobility restrictions (e.g., lockdowns, closures of borders, reductions on the economic activity), and their epidemiological and socioeconomic impact depend precisely on the spatial and temporal scales of deployment.

While RMT techniques are only meaningful for large-sized and sufficiently random systems, they allow us to obtain factual estimates for the epidemic thresholds even with limited data availability, as our results depend on simple summary statistics. Based on these, we can identify the parameters relevant for disease spread in a range of epidemic scenarios (e.g., vaccination, new disease strains, non-pharmaceutical interventions). Assuming that epidemiological and connectivity parameters are inherently random quantities makes our results robust to natural stochastic variability. First, the epidemic thresholds we obtain are deterministic, and second, great accuracy is found even for small systems.

Future investigations on this topic should involve more complex human mobility and connectivity patterns, incorporating further spatial and temporal structure into the networks. This would introduce more social realism in our framework. For example, commuting should be spatially restricted to relatively close geographical areas, and migratory patterns could follow seasonal trends, incorporating the temporal heterogeneity of human mobility as well. Further extensions of our model should include the inherent stochasticity in disease transmission, both between and within the patches. It is known that the configuration of human contact patterns may change the initial spread of an infectious disease^67^. Indeed, the homogeneous mixing assumed within the patches is a limiting assumption that may be restricting the generality of the conclusions reached here. It would thus be desirable to compare our findings with models grounded in other frameworks, such as agent-based and/or stochastic models. However, combining our random spatial formalism with a stochastic disease transmission model may entail nontrivial challenges, in particular for the derivation of theoretical results. The study of the interplay between these two types of randomness seems like an exciting future perspective able to better capture realistic human behaviour and increase our preparedness to contain the spread of infectious diseases. Future work also includes adjusting our model to real mobility and disease transmission data, available after the COVID-19 pandemic, to test the possible uses of this framework as a real-world tool for risk estimation and factual prediction.

## Methods

### Metapopulation SIR model

We study a metapopulation SIR model, with each local population representing a node in a network with two types of mobility along its edges. The infected subsystem of the model consists of *N* differential equations describing the temporal evolution of the number of infected individuals at each of the nodes of the network:

3 $$\begin{aligned} \begin{aligned} \frac{dI_i}{dt}&= \beta _i \frac{S_i}{N_i}I_{i} - (\alpha _{i}+d_{i})I_i + \sum _{j=1}^{N} \beta _{j}c_{ji}\frac{S_{i}}{N_{i}}I_{j} - \sum _{j=1}^N m_{ji} I_i + \sum _{j=1}^N m_{ij} I_j, \end{aligned} \end{aligned}$$

where $i\in \{1,\dots ,N\}$ and $N_{i} = S_{i}+I_{i}+R_{i}$ denotes the total population at node *i*, obtained as the sum of susceptible, infected and recovered individuals. The parameters $\beta _{i}$ and $\alpha _{i}$ denote the transmission and recovery rates of the disease at node *i*, and $d_{i}$ denotes the death rate of the population at the node. We assume that no disease-related deaths occur, and denote by $\gamma _{i} = \alpha _{i}+d_{i}$ the combined rate of depletion of infected individuals. As explained above, the coefficients $c_{ij}$ and $m_{ij}$ model the connectivity between the nodes of the network and are drawn from two arbitrary probability distributions. More precisely, we let *c* be a positive random variable with mean and variance $\mu _{c}$ and $\sigma _{c}$, and draw each coefficient $c_{ij}$ in (3) from this random variable. New infections are then generated at patch *i* from interactions of susceptible individuals from the patch with infected individuals from other patches *j* at a rate $\beta _{j}c_{ji}$. This force of infection may be associated to the movement of individuals visiting other patches and returning to their origin patch, which may differ in strength from that generated at their original location. Analogously, we let *m* be a positive random variable following certain distribution with mean $\mu _{m}$ and standard deviation $\sigma _{m}$ from which each coefficient $m_{ij}$ is drawn. These parameters model the per-capita movement rates of individuals that go from patch *j* to patch *i* and stay there. We assume that $c_{ii}=0$ $m_{ii}=0$ for all *i* in (3). In general, we will be interested in networks in which the number of patches *N* is large.

The conditions $I_{i} = R_{i} = 0$ for all *i* characterize the disease-free equilibrium (DFE) of the system, a steady state in which no infected population exists; its explicit expression can be found by solving the system of equations

4 $$\begin{aligned} -\sum _i m_{ji} S_i^{*} + \sum _j m_{ij} S_j^{*} = 0, \qquad i\in \{1,\dots ,N\}. \end{aligned}$$

The migration flows determine the distribution of the population of the system across patches at the DFE, as the local outgoing and incoming flows need to compensate in order for each local population to be constant. We are interested in evaluating whether a small number of infectious individuals in a susceptible population will proliferate (potentially resulting in an epidemic scenario) or will decay and eventually disappear. In dynamical systems terminology, this is equivalent to studying the stability of the infected subsystem Eq. (3) around the DFE characterized by Eq. (4). To do so, one computes the rightmost eigenvalue of the corresponding Jacobian matrix, as this quantity measures the initial exponential growth rate of the disease in its simplified linearized dynamic behaviour^51^.

### Random matrices

A central idea in our approach is to understand the Jacobian matrix of the system around the DFE as an algebraic perturbation of a random matrix, closely related to another random matrix which eigenvalue distribution can be accurately predicted for large systems. Schematically, we perform the following decomposition of the Jacobian of the linearized infected subsystem around the DFE

5 $$\begin{aligned} \begin{aligned} Jacobian&= \textit{random matrix} \\&= (\textit{structure matrix}) + (\textit{centered noise}) + (\textit{multiple of identity}), \end{aligned} \end{aligned}$$

where the *structure* matrix is assumed to be deterministic and the entries of the *noise* matrix have mean zero. To find the eigenvalues of the Jacobian, one is really interested in computing those of the $structure + noise$ matrix, as adding a multiple of the identity results in a translation of the eigenvalues of the original matrix (note that $\det {\left( A+k Id\right) } = k+\det {A}$)). They can be found using a theorem of Tao^52^, that generalizes the well-known circular law from random matrix theory^54^ to low-rank perturbations of random matrices. Loosely speaking, the resulting asymptotic eigenvalue distribution consists of two components: the bulk of the eigenvalues, that distribute uniformly on a circle which size depends only on the parameters of the *noise* matrix, together with a small number of outlier eigenvalues that depend on the *structure* matrix. This result holds as long as the rank of the *structure* matrix is low compared to its size (*o*(*N*), where *N* is the size of the matrices in Eq. (5)).

For instance, assuming that the transmission and depletion rates $\beta _{i}$ and $\gamma _{i} = \alpha _{i}+d_{i}$ are the same across the nodes of the system (we drop the subscript *i* accordingly), the Jacobian of the infected subsystem of the model Eq. (3) at DFE reads

6 $$\begin{aligned} J = \begin{pmatrix} \beta -\gamma - \sum _{j}m_{j1}&{} \beta c_{21} + m_{12}&{} \beta c_{31} + m_{13} &{} \dots &{} \beta c_{N1} + m_{1N} \\ \beta c_{12}+ m_{21} &{} \beta - \gamma - \sum _{j}m_{j2}&{} \beta c_{32}+ m_{23} &{} \dots &{} \beta c_{N2} + m_{2N} \\ \beta c_{13} + m_{31} &{} \beta c_{23} + m_{32}&{} \beta - \gamma - \sum _{j}m_{j3} &{} \dots &{} \beta c_{N3} + m_{3N}\\ &{} &{} &{} &{} \\ \beta c_{1N} + m_{N1} &{} \beta c_{2N} + m_{2N}&{}\beta c_{3N} + m_{N3}&{} \dots &{} \beta - \gamma - \sum _{j}m_{jN} \end{pmatrix}. \end{aligned}$$

**Figure 6:**
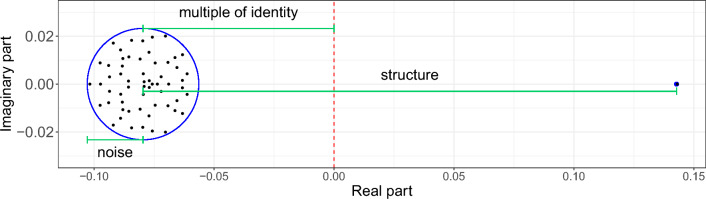
Eigenvalue distribution for the Jacobian matrix $J'$. The location of the eigenvalues of $J'$ depends on each of the three matrices given in (7): the size of the circle on which the bulk of the eigenvalues is uniformly distributed depends on the noise matrix, the distance between its center and the origin depends on the multiple of the identity, and the distance between its center and the single outlier eigenvalue depends on the structure matrix.

As we argue in Sect. 1.1 of the Supplementary Information, the eigenvalue distribution of this matrix coincides with that of the following one

7 $$\begin{aligned} J' = \underbrace{(\beta \mu _{c} + \mu _{m}) \mathbbm{1}_{N}}_{\textit{structure matrix}} \; + \negthickspace \underbrace{\mathcal {G}_{N}(0,\sigma )}_{\textit{centered noise}} \negthickspace + \; \underbrace{(\beta - \gamma - \beta \mu _{c} - N\mu _{m})I_{N}}_{\textit{multiple of identity}}, \end{aligned}$$

where $\mathbbm{1}_{N}$ is a $N\times N$ matrix of ones, the $N\times N$ matrix $\mathcal {G}_{N}(0,\sigma )$ has iid entries with mean zero and standard deviation $\sigma$, and $I_{N}$ denotes the identity matrix of size *N*. Let us recall that the commuting and migration rates are drawn from random variables with mean and standard deviation $(\mu _{c},\sigma _{c})$ and $(\mu _{m},\sigma _{m})$ respectively. We have denoted in (6) the variance of the off-diagonal entries of the Jacobian by

8 $$\begin{aligned} \sigma ^{2} = \beta ^{2}\sigma _{c}^{2} + 2\beta \tau +\sigma _{m}^{2}, \end{aligned}$$

which results from a combination of the variance of *c* and *m* (we denote by $\tau$ the covariance between the commuting and migration coefficients in (8)). Using theorem 2.8 in O’Rourke and Renfrew (which includes a convenient generalization of the circular law, as explained in Supplementary Sect. 1.2), we find that for large *N* the bulk of the eigenvalues of $J'$ almost surely lie on the circle of radius $\sigma \sqrt{N}$ centered at $\beta (1-\mu _{c})-\gamma -N\mu _{m}$. The structure matrix has rank one and generates a single outlier eigenvalue at $\beta \mu _{c}(N-1)+\beta -\gamma$ (see Fig. 6 for a schematic representation). The epidemic threshold (2) follows precisely from restricting this eigenvalue to have strictly negative real part. For our model, there is no need to control the location of the bulk of the eigenvalues, as for the parameter range of interest this always lies on the left side of the outlier. More details related to this phenomenon and other technical aspects of this reasoning are provided in Sect. 1.1 of the Supplementary Information. Interestingly, we see that the only statistical properties of the random variables *c* and *m* that influence the eigenvalue distribution of the Jacobian matrix are their mean and variance.

### Perturbations

A similar reasoning provides analytical expressions for the outlier eigenvalues of the several generalizations of the model described in Results. For these scenarios, a different structure matrix is chosen in Eq. (7) to appropriately model each of the modifications of the base system considered in the text. We display in Supplementary Sects. 2 and 3.1 the appropriate choices of structure matrix, which lead to the following generalizations of the epidemic threshold *s*(*J*) upon using the low rank perturbation theorem^53^ again. We assume below that *k* is small compared to the size of the network *N* in order for the low-rank perturbation theorem^53^ to hold, and recall that the average of the commuting and migration coefficients are denoted by $\mu _{c}$ and $\mu _{m}$ respectively.

- Perturbed infectiousness scenarios: transmission rate changes from $\beta$ to $\beta ^{*} = \beta +\alpha$ at *k* nodes of the network
  9 $$\begin{aligned} s(J)&=\frac{N}{2}\left( \beta \mu _{c} + \mu _{m}\right) + \frac{\alpha }{2}\left( 1+(k-1)\mu _{c}\right) + \beta (1-\mu _{c}) - \gamma - N\mu _{m} \nonumber \\&\quad +\frac{1}{2}\sqrt{N^{2}\left( \beta \mu _{c} + \mu _{m}\right) ^{2} + \alpha ^{2}\left( 1+(k-1)\mu _{c}\right) ^{2} + 2\alpha \left( \beta \mu _{c}+\mu _{m}\right) \left[ N\left( 1+(k-1)\mu _{c}\right) + 2(N-k)\left( \mu _{c}-1\right) \right] }. \end{aligned}$$
- Strategy A (resp. B) for mobility reduction: commuting flows change from $c_{ij}$ to $\mu _{c}+\nu$ for all outgoing (resp. incoming) flows at *k* nodes of the network (in the main article, $\nu =-\mu _{c}$)
  10 $$\begin{aligned} s(J)&= \frac{N}{2}\left( \beta \mu _{c}+\mu _m\right) + \frac{k-1}{2}\beta \nu +\beta \left( 1-\mu _{c}\right) -\gamma - N\mu _{m} \nonumber \\&\quad +\frac{1}{2}\sqrt{N^{2}\left( \beta \mu _{c}+\mu _m\right) ^2 + (k-1)^2\beta ^2\nu ^2 + 2(N+Nk-4k)\left( \beta \mu _{c}+\mu _m\right) \beta \nu }. \end{aligned}$$
- Strategy C for mobility reduction: commuting flows change from $c_{ij}$ to $\mu _{c}+\nu$ for all incoming and outgoing flows at *k* nodes of the network (in the main article, $\nu = -\mu _{c}$)
  11 $$\begin{aligned} s(J)&= \frac{N}{2}\left( \beta \mu _{c}+\mu _m\right) + \frac{k-1}{2}\beta \nu +\beta \left( 1-\mu _{c}\right) -\gamma -N\mu _{m} \nonumber \\&\quad + \frac{1}{2}\sqrt{N^{2}\left( \beta \mu _{c}+\mu _m\right) ^2 + 2\beta \nu (\beta \mu _{c}+\mu _{m})(N+k(3N-2k-2)) + \beta ^2\nu ^2\left( 4k(N-k)+(k-1)^2 \right) }. \end{aligned}$$

For Strategies D, E and F, it is the noise matrix that needs to be chosen differently in (7) in order to model the different mobility reductions. Due to the universality phenomenon in random matrix theory (only the moments of up to second order of the random variables *c* and *m* influence the eigenvalue distribution of the Jacobian matrix, not their particular shape) and the parameter range for the coefficients of the system (correlations $\rho$ between flows going in opposite directions do not influence the stability of the system, only the bulk of the eigenvalues), we find a common expression for the three strategies.

- Strategies D, E and F for mobility reduction: mean of the commuting coefficients $c_{ij}$ over the whole network changes from $\mu _{c}$ to $\mu _{c}+\nu '$ (in the main article, $\nu ' = -\mu _{c}k/N$)
  12 $$\begin{aligned} s(J) = (N-1)\beta (\mu _{c}+\nu ') + \beta - \gamma . \end{aligned}$$

### Correlated flows

The epidemic threshold *s*(*J*) is modified as well when allowing for the mobility flows to be non-trivially correlated. Following Baron et al.^59^, we introduce the following correlations between the mobility coefficients in matrix (6) that share a common index

13 $$\begin{aligned} \begin{aligned} \rho _{c}&= \text {Cor}(c_{ij},c_{ji}),&\qquad \rho _{m}&= \text {Cor}(m_{ij},m_{ji}),\\ \Gamma _{c}&= \text {Cor}(c_{ij},c_{ki}),&\qquad \Gamma _{m}&= \text {Cor}(m_{ij},m_{ki}),\\ r_{c}&= \text {Cor}(c_{ij},c_{ik}),&\qquad r_{m}&= \text {Cor}(m_{ij},m_{ik}), \\ c_{c}&= \text {Cor}(c_{ji},c_{ki}),&\qquad c_{m}&= \text {Cor}(m_{ji},m_{ki}). \end{aligned} \end{aligned}$$

These correlations can be understood as involving mobility flows on the same edge going in opposite directions ($\rho$), incoming and outgoing flows at the nodes of the network ($\Gamma$), incoming flows at the nodes of the network (*r*) and outgoing flows at the nodes of the network (*c*). The subscripts in (13) denote the random variable involved in the corresponding expression. Examples of networks displaying these correlations are shown in Supplementary Sect. 3.3. It is explained in Ref.^59^ that no correlations between other pairs of elements of the matrices are relevant in the asymptotic regime. The corresponding parameters for the elements of the random Jacobian matrix (6) result from a combination of those of the commuting and migration networks

14 $$\begin{aligned} \begin{aligned} \sigma&= \sqrt{\beta ^{2}\sigma _{c}^{2} + 2\beta \tau + \sigma _{m}^{2}}, \\ \rho&= (\rho _{c}\beta ^{2}\sigma _{c}^{2} + \rho _{m}\sigma _{m}^{2})/\sigma ^{2}, \\ \Gamma&= (\Gamma _{c}\beta ^{2}\sigma _{c}^{2} + \Gamma _{m}\sigma _{m}^{2})/\sigma ^{2}, \\ r&= (r_{c}\beta ^{2}\sigma _{c}^{2} + r_{m}\sigma _{m}^{2})/\sigma ^{2}, \\ c&= (c_{c}\beta ^{2}\sigma _{c}^{2} + c_{m}\sigma _{m}^{2})/\sigma ^{2}, \end{aligned} \end{aligned}$$

where $\tau$ denotes the covariance between the commuting and migration flows connecting the same pair of nodes ($\tau = \text {Cov}(c_{ij},m_{ij})$). We have assumed in (14) that all covariances involving other pairs of commuting and migration coefficients are zero for simplicity; the more general case follows analogously. After a rescaling on Baron *et al*’s result (see Supplementary Sect. 3.3), we find that the outlier eigenvalue of the system is now located at

15 $$\begin{aligned} s(J) = \beta \mu _{c}(N-1) + \beta - \gamma + \frac{N}{2}\left( \beta \mu _{c} + \mu _{m} \right) \left( 1+\frac{\rho }{N\Gamma } \right) \left( \sqrt{1+\frac{4\Gamma \sigma ^2}{(\beta \mu _{c}+\mu _{m})^{2}}}-1 \right) . \end{aligned}$$

Note that this expression coincides with the epidemic threshold of the uncorrelated case Eq. (2), with the addition of the last term in the sum above. Moreover, we find that *s*(*J*) now depends on the average migration rate $\mu _{m}$ and the joint variance of the network $\sigma ^{2}$. In particular, the sign of $\Gamma$ determines whether the threshold Eq. (15) is higher or lower than in the uncorrelated case (see Fig. 5), as only the last parenthesis in this expression may take negative values (variations caused by the second-to-last parenthesis vanish in the asymptotic regime; in particular its effect on the stability is negligible for sufficiently large values of *N*; see Supplementary Fig. 16).

## Supplementary Information

Supplementary Information.

## Data Availability

The datasets used and/or analysed during the current study available from the corresponding author on reasonable request.
