# Supplementary material for: Epidemic thresholds and human mobility
Source: Sci Rep. 2023 Jul 14;13:11409. doi: 10.1038/s41598-023-38395-0 (PMC10349094; doi:10.1038/s41598-023-38395-0)
Supplement: Supplementary file 1 — Supplementary Information. [file 41598_2023_38395_MOESM1_ESM.pdf]

Supplementary Information
for
"Epidemic thresholds and human mobility"

Marta Pardo-Araujo[1,†], David García-García[2,3,†,*], David Alonso[1], Frederic Bartumeus[1,4,5]

[1]Centre d'Estudis Avançats de Blanes (CEAB-CSIC, Blanes, Spain)

[2]Consorcio de Investigación Biomédica en Red de Epidemiología y Salud Pública (CIBERESP, Madrid, Spain)

[3]Centro Nacional de Epidemiología (CNE-ISCIII, Madrid, Spain)

[4]Institució Catalana de Recerca i Estudis Avançats (ICREA, Barcelona, Spain)

[5]Centre de Recerca Ecològica i Aplicacions Forestals (CREAF, Barcelona, Spain)

[†]These authors contributed equally

[*]Corresponding author

Contents

1 A SIR metapopulation model **2**
- 1.1 Random matrices . . . 6
- 1.2 Next-generation matrix . . . 11
- 1.3 Gershgorin and Perron-Frobenius theorems . . . 12

2 Transmission and disease distribution **14**

3 Random mobility networks **18**
- 3.1 Restrictions in commuting flows . . . 20
- 3.2 Perturbed migration scenarios . . . 23
- 3.3 Generally correlated networks . . . 23

1 A SIR metapopulation model

We consider a susceptible-infected-recovered (SIR, [15, 3, 8]) model describing the spread of an infectious disease over a discrete metapopulation, consisting of N patches connected by a weighted network. At each patch i, individuals remain at three possible states: susceptible, infected or recovered (denoted by S_i, I_i and R_i respectively). These populations evolve according to the following system of differential equations

$$\begin{aligned}
\frac{dS_i}{dt} &= \Lambda_i N_i - \beta_i \frac{S_i}{N_i} I_i - d_i S_i + \theta_i R_i - \sum_{j=1}^{N} \beta_j c_{ji} \frac{S_i}{N_i} I_j - \sum_{j=1}^{N} m_{ji} S_i + \sum_{j=1}^{N} m_{ij} S_j, \\
\frac{dI_i}{dt} &= \beta_i \frac{S_i}{N_i} I_i - (\alpha_i + \delta_i + d_i) I_i + \sum_{j=1}^{N} \beta_j c_{ji} \frac{S_i}{N_i} I_j - \sum_{j=1}^{N} m_{ji} I_i + \sum_{j=1}^{N} m_{ij} I_j, \qquad (1) \\
\frac{dR_i}{dt} &= \alpha_i I_i - (\theta_i + d_i) R_i - \sum_{j=1}^{N} m_{ji} R_i + \sum_{j=1}^{N} m_{ij} R_j,
\end{aligned}$$

where $i \in \{1, \dots, N\}$ and $N_i = S_i + I_i + R_i$ denotes the total population at patch i. Table 1 contains a description of the epidemiological parameters of the system. These parameters model the usual natural dynamics of human populations (births and deaths), as well as the life cycle of the disease (infection, recovery, loss of immunity). In addition to these, there are two qualitatively different terms in Eq. (1) that model the mobility of individuals between patches.

Parameter	Description
Λ_i	Birth rate
β_i	Transmission rate of the disease
d_i	Natural death rate (excluding the modeled disease)
δ_i	Disease-related death rate
θ_i	Rate of loss of immunity to the disease
α_i	Rate of disease overcome

Table 1: **Biological parameters of the model.** Rates describing the natural dynamics of the population and the life cycle of the disease at patch i of the system (Eq. (1)).

The first of these terms corresponds to the short-term mobility between patches, and is labelled as *commuting* throughout the article. It models the infections generated at each patch i derived from interactions of susceptible individuals from patch i with infected individuals from other patches j. We assume that these new infections are generated at a rate $\beta_j c_{ji}$, that factors the transmission rate at patch j and a corrective factor c_{ji}. Interactions may occur anywhere in the network and the corresponding individuals return to their patch of origin. We assume that the reference transmission rate is the one associated to the patch of origin j of the infected individual. The second term associated to human mobility concerns movement between patches with no return, and is labelled as *migration*. We assume that individuals go from patch j to patch i at a rate m_{ij} and do not go back to their patch of origin. Depending on the chosen timescale for the system, this could represent daily or seasonal flows between locations in the network, for instance.

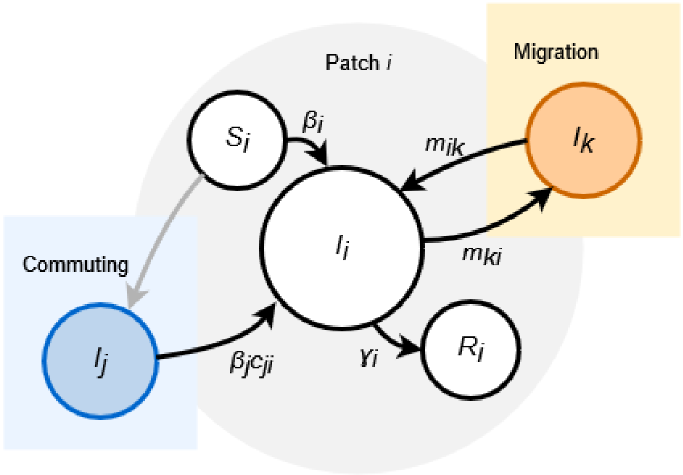

Figure 1: **The model.** Three different processes may modify the number of infected individuals at each patch i. First, the natural dynamics of the disease, consisting of contagions of susceptible from infected individuals from the patch (at a rate β_i of transmission of the disease) and depletion of infected individuals (at a rate γ_i, that combines the rate of true recovery from the disease and the natural death rate of the population). Second, contagions of susceptible from infected individuals from other patches j (at a rate $\beta_j c_{ji}$, that factors the local disease transmission rate β_j and a corrective term c_{ji} quantifying the force of infection on patch i generated by interactions with individuals from patch j). Third, arrivals/departures of infected individuals from/to other patches j (at rates m_{ij} and m_{ji} respectively).

As mentioned in the text, both our commuting and migration terms are standard in metapopulation epidemic models [14, 23], and are included to display the potential of RMT techniques in this context. Our choice of model is particularly suited for including spatial heterogeneities in transmission, such as disease strains, vaccination, or non-pharmaceutical interventions, but our methods could be developed analogously for other variations on the modelling of the transmission events (see for instance [18, 22, 17, 4, 30]).

Rather than choosing specific values for the coefficients c_{ij} and m_{ij} in Eq. (1), we assume that these are drawn from probability distributions. More precisely, we assume that every coefficient c_{ij} is a realization of an iid random variable following a certain positive distribution c, with given mean and variance (μ_c, σ_c^2). Analogously, we assume that the coefficients m_{ij} are drawn from iid random variables following another given positive distribution m, with mean and variance (μ_m, σ_m^2). As we show in Section 1.1 below, these two parameters (mean and variance of the distributions) are the only ones relevant for the possibility of spread of the disease over the system. No further constraints are assumed for the distributions, besides their positiveness. From a theoretical point of view, this flexibility should allow us to capture real-world mobility networks in our model, by simply computing the mean and variance of the empirically observed connectivity flows between the locations considered in the system. We incorporate nevertheless in Section 3 some structural variations in the network and show how these may affect the spread of the disease.

We will focus on the computation of epidemic thresholds, that determine the initial growth of the disease. These depend on the transmission and recovery rates of the disease and the mobility features of the network. Numerical simulations show that the remaining epidemiological and biological parameters in Eq. (1) influence instead the long-term behaviour of the system (Fig. 2). For instance, even an unstable scenario in which the infected population grows initially may result in a non-endemic disease if the disease-related death rate is high enough. In general, increasing the mortality rates produces an instant decay in the number of infected individuals, while if the natural death rate is lower than the birth rate and the system is unstable then the number of infected individuals will grow exponentially. An increase on θ, the rate of loss of immunity, results in a larger number of infected individuals at the endemic state of the disease. On the contrary, rising the rate of disease overcome α causes an endemic state with a smaller number of infected individuals. In both cases, the qualitative behaviour of the outbreak remains the same (Fig. 2).

In the following, we assume that the system in Eq. (1) is closed, that is, that the total population remains constant over time. This is a standard assumption in epidemic models for diseases with a relatively low death rate during their initial growth period [10]. This condition is achieved by assuming equal birth and natural death rates across patches ($\Lambda_i = d_i$ for all i), and no disease-related mortality ($\delta_i = 0$ for all i). We denote the resulting recovery rate as $\gamma_i = \alpha_i + d_i$ here and in the main article, combining the true recovery rate and the natural death rate. Under these hypothesis, the domain $\mathbb{R}_+^{3N}$ is an invariant subspace under the action of the dynamical system (Eq. (1)), and thus the model is well-defined. This means in particular that if the initial conditions of the system are biologically feasible, then the system will remain to be such throughout time (see for instance Proposition 2 in [25] for a similar model).

Another consequence of the closedness of the system is that the disease-free state becomes in fact an equilibrium point of the dynamical system. The conditions $I_i = R_i = 0$ for all i characterize the disease-free equilibrium (DFE) of the system Eq. (1), that takes the form $(S_1^*, 0, 0, S_2^*, 0, 0, \dots)$. Its explicit expression can be found by solving the system of equations (see Theorem 3.3 in [4])

$$-\sum_i m_{ij} S_i^* + \sum_j m_{ij} S_j^* = 0, \qquad i \in \{1, \dots, N\}. \tag{2}$$

That is, the distribution of the migration flows determines the distribution of the population over the patches of the system at the DFE, as the outgoing and incoming flows need to compensate in order for each local subpopulation to be constant. Note that the system (1) models the simplifying assumption that migration rates are independent of the state of the individuals.

The rightmost eigenvalue of the Jacobian matrix of the system at the DFE, $s(J)$, governs the possibility of spread of the disease [9, 10]. As follows from classical dynamical systems theory, if there is any eigenvalue with real part greater than zero the system is unstable at the DFE, i.e., the number of infected individuals tends to grow initially. If all the eigenvalues of the Jacobian at the DFE have negative real part, the system is stable and the disease dies out upon the introduction of a small number of infected individuals in the network. This behaviour provides the threshold condition analogous to that of R_0 exploited over the text. Moreover, the magnitude of the total

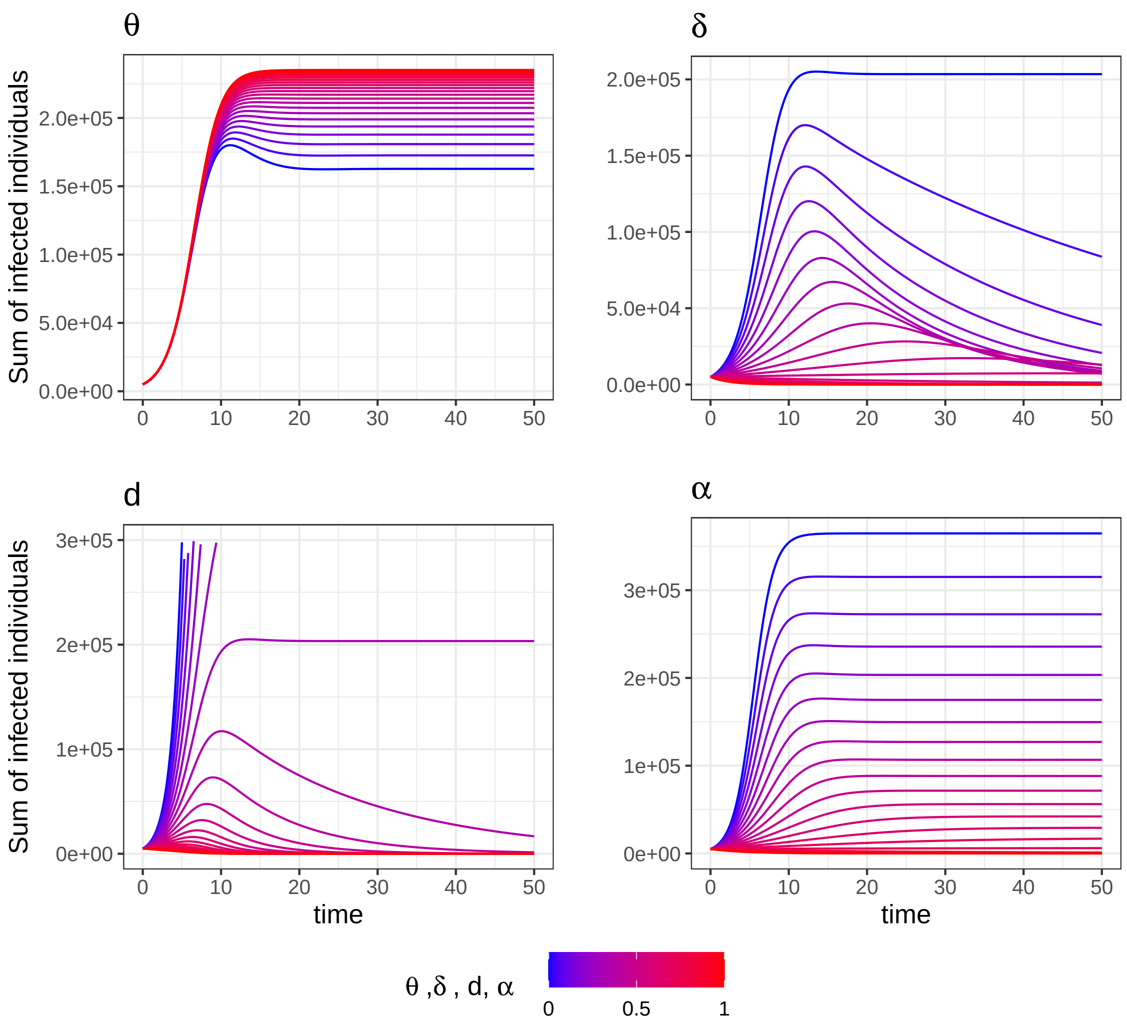

Figure 2: **Rates of recovery, death and loss of immunity.** Total number of infected individuals for a system with $\beta = 0.1$ over time for several values of the recovery rate α, the natural death rate d, the disease-related death rate δ, and the recovery rate θ. All rates are assumed to coincide across patches and the system is unstable in all cases, verifying $s(J) > 0$.

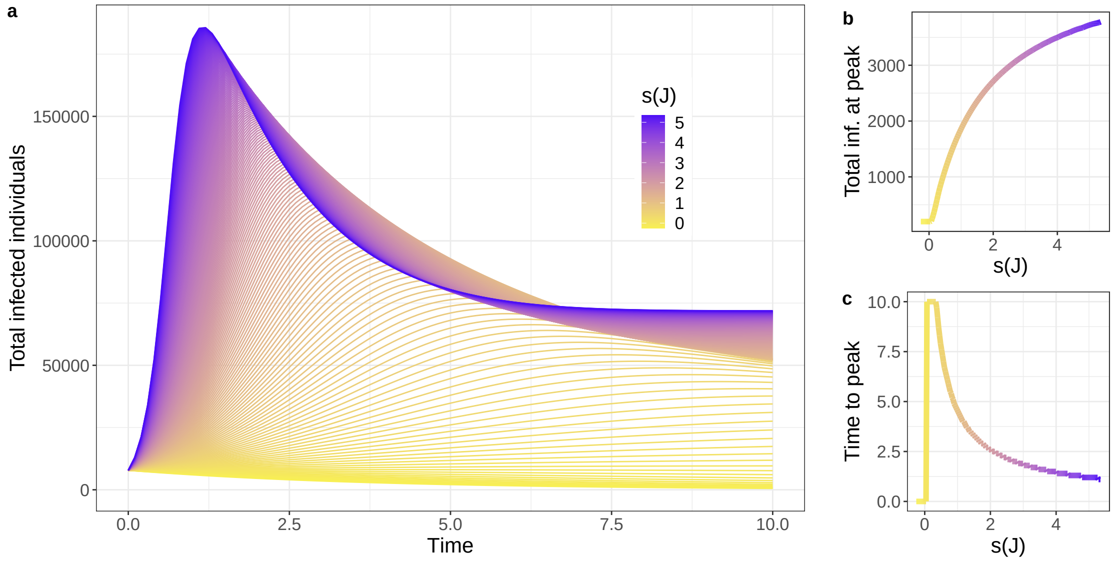

Figure 3: **The epidemic threshold $s(J)$ shapes the evolution of the disease.** Total number of infected individuals over the system, maximum of infected individuals at a single patch, and time when this maximum is attained in terms of the epidemic threshold $s(J)$. Higher values of $s(J)$ result in an earlier and steeper epidemic.

number of infected individuals over the system, the maximum of infected individuals at a single patch, and the time to arrive to the epidemic peak also depend non-linearly on the epidemic threshold $s(J)$ (see Fig. 3 for some examples). Let us note that, unlike for systems modelling ecological communities [2, 5], the differential equations for the susceptible, infected and recovered populations in (1) are coupled; it is only due to the known properties of SIR-like models [10] that the subsequent analysis by means of the Jacobian of the simpler infected subsystem is possible.

1.1 Random matrices

We assume for the moment that the rates of disease transmission and recovery of infected individuals are the same at all the nodes of the network, and denote them by β and γ respectively. Under these conditions, the Jacobian matrix of the infected subsystem of the model (1) at DFE reads as follows

$$J = \begin{pmatrix} \beta - \gamma - \sum_j m_{j1} & \beta c_{21} + m_{12} & \beta c_{31} + m_{13} & \dots & \beta c_{N1} + m_{1N} \\ \beta c_{12} + m_{21} & \beta - \gamma - \sum_j m_{j2} & \beta c_{32} + m_{23} & \dots & \beta c_{N2} + m_{2N} \\ \beta c_{13} + m_{31} & \beta c_{23} + m_{32} & \beta - \gamma - \sum_j m_{j3} & \dots & \beta c_{N3} + m_{3N} \\ \vdots & \vdots & \vdots & & \vdots \\ \beta c_{1N} + m_{N1} & \beta c_{2N} + m_{2N} & \beta c_{3N} + m_{N3} & \dots & \beta - \gamma - \sum_j m_{jN} \end{pmatrix}. \quad (3)$$

Recall that the commuting and migration coefficients are drawn from random variables with mean and standard deviation (μ_c, σ_c) and (μ_m, σ_m), respectively. As we argue below, the asymptotic eigenvalue distribution of J coincides with that of

$$J' = \underbrace{(\beta\mu_c + \mu_m)\mathbb{1}_N}_{\text{structure matrix}} + \underbrace{\mathcal{G}_N(0,\sigma)}_{\text{centered noise}} + \underbrace{(\beta - \gamma - \beta\mu_c - N\mu_m)I_N}_{\text{multiple of identity}}, \tag{4}$$

where $\mathbb{1}_N$ is a $N \times N$ matrix of ones, the $N \times N$ matrix $\mathcal{G}_N(0,\sigma)$ has iid entries following a certain distribution with mean zero and standard deviation $\sigma = \sqrt{\beta^2\sigma_c^2 + 2\beta\tau + \sigma_m^2}$ (where $\tau = \sigma_c\sigma_m \mathrm{Cor}(c_{ij}, m_{ij})$), and I_N denotes the identity matrix of size N. We have chosen the monikers in Eq. (4) to help identify the role of each matrix in the eigenvalue distribution of J': the *structure* matrix provides the mean of the off-diagonal entries of J', the *noise* matrix captures their fluctuations around this mean, and the *multiple of identity* sets the mean of the diagonal.

We use the low rank perturbation theorem of Tao [26, 21], that generalizes the well-known circular law from RMT [27], providing the asymptotic eigenvalue distribution of matrices such as J' whenever the structure matrix has low rank. Using this result (we actually use theorem 2.8 in [21], as we explain below), we find that the eigenvalue distribution of J' consists on two regions in the asymptotic $N \to \infty$ regime. These are a circle on the complex plane on which the majority of the eigenvalues are uniformly distributed (the bulk), and a single outlier eigenvalue located on the real axis. After rescaling these distributions in N to match our model (see below for technical details), we find that the circle has radius $\sigma\sqrt{N}$ and is centered at $\beta(1-\mu_c) - \gamma - N\mu_m$, and that the outlier eigenvalue is located at $\beta\mu_c(N-1) + \beta - \gamma$.

We now provide technical details that justify why the eigenvalue distribution of the original Jacobian matrix J given in Eq. (3) coincides in the asymptotic regime with the one of J':

- A central phenomenon in RMT is the universality of the asymptotic eigenvalue distributions of random matrices [27, 12]: eigenvalues of matrices belonging to a large class of distributions share a common macroscopic behaviour in the large-N limit, that only depends on the mean and variance of the entries of the random matrix and not on the particular distribution followed by these. This is true for the circular law and also for the generalizations of it used throughout the article. As a consequence, the limiting distribution of the eigenvalues of J coincides with that of matrix J' for any choice of *noise* matrix, as long as its entries have the same variance σ^2. Said differently, the eigenvalues of J follow the same asymptotic distribution for any choice of mobility coefficients having the same mean and variance and regardless of their particular values.

- Rigorous statements of the circular law [27], the low-rank perturbation theorem [26] and theorem 2.8 in [21] involve a different scaling of the random matrix J', providing a fixed limiting distribution for the bulk of the eigenvalues. The matrix J' chosen here results in a rescaling of the distribution given by the low-rank perturbation theorem, after the substitutions $\mu \to \sqrt{N}\mu$, $\sigma \to \sqrt{N}\sigma$ (for example in theorem 2.8 in [21]). The obtained distributions are also accurate in this case, as has been observed in previous works [2, 1, 13, 24]. One of the advantages of RMT results is that often asymptotic convergence is apparent already at small values of N, and in particular we observe that is the case after this rescaling. See Fig. 4 for some examples of this fact, that holds for J as well.

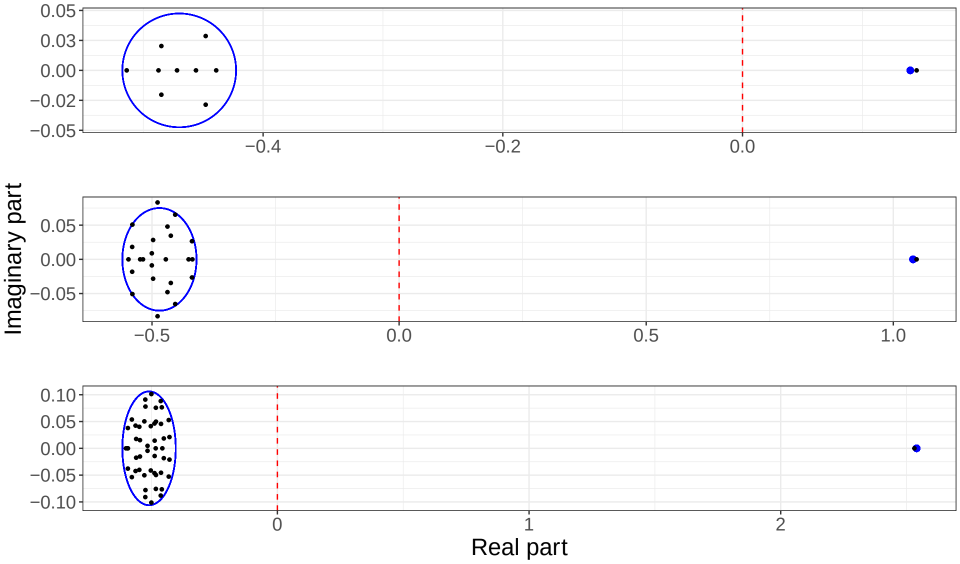

Figure 4: **Convergence to the circular law for low values of N.** The convergence of the eigenvalue distributions of random matrices to their predicted locations is apparent even for small values of N. From top to bottom, the plots show the eigenvalues of systems of sizes $N = 10, 25, 50$. The off-diagonal entries of the Jacobian have mean $\beta\mu_c + \mu_m = 0.045$ and standard deviation $\sigma = 0.01$ and the transmission and recovery rates verify $\beta - \gamma = -0.4$. Note that the predicted distribution is accurate for the outlier as well, even after the re-scaling in N explained in the text.

- Another difference between J and J' is that their diagonal elements are only equal in mean. O'Rourke and Renfrew prove [21] that the limiting eigenvalue distribution provided by the circular law is independent of the variance of the diagonal entries of the matrix, as long as this is finite. Note, however, that the results of RMT hold asymptotically for matrices with fixed parameters, and the variance of the diagonal entries of J grows with N. Numerical experiments show nevertheless a very close resemblance between the empirically computed eigenvalues of J and their theoretical expected distribution whenever σ_m is relatively small. Even for larger values of this parameter, that controls the variance $(N-1)\sigma_m^2$ of the diagonal entries of J, the location of the outlier eigenvalue seems to be accurately predicted by the low-rank perturbation theorem, while the circular distribution of the bulk of the eigenvalues is deformed (see Fig. 5). For simplicity, we consider throughout the article parameter values for the random matrices such that the variance of their diagonal elements does not cause a significant perturbation of the circular law; these seem to match realistic assumptions on human mobility.

As explained in section 1, the system will be stable whenever all the eigenvalues of the Jacobian matrix J lie on the left half of the complex plane, and therefore one should in principle control for both the bulk region and the outlier eigenvalue to verify this condition. This results in the following two conditions for the stability of the system

$$\beta\mu_c(N-1) + \beta - \gamma < 0 \tag{5}$$

$$\beta(1-\mu_c) - \gamma - N\mu_m + \sigma\sqrt{N} < 0 \tag{6}$$

However, we see that if the first condition holds but the second is violated, then

$$\beta\mu_c(N-1) < \gamma - \beta < \sigma\sqrt{N} - \beta\mu_c - N\mu_m, \tag{7}$$

or, equivalently, that the coefficient of variation of the off-diagonal entries of J verifies

$$\sqrt{N} < \frac{\sigma}{\beta\mu_c + \mu_m}. \tag{8}$$

Note, however, that the the off-diagonal entries of J follow a positive distribution, as explained in section 1. Without loss of generality, if we assume this distribution to be compactly supported on an interval $[0, R]$ for some $R > 0$, the Bhatia-Davies inequality [7] introduces another constraint on the size of its variance

$$\sigma^2 \leq (R-\mu)\mu, \tag{9}$$

where we have denoted by $\mu = \beta\mu_c + \mu_m$ the mean of the off-diagonal entries of J. Combining Eqs. (8) and (9), we see that in order for the first stability condition to hold and for the second to be violated, the distribution of the off-diagonal entries of J would need to verify

$$\mu(N+1) = (\beta\mu_c + \mu_m)(N+1) < R. \tag{10}$$

In the asymptotic regime, this results in a contradiction, and even for finite but moderately large values of N this results in highly degenerated distributions that do not seem to model realistic systems. This means that for practical purposes, the outlier eigenvalue always lies on the right of

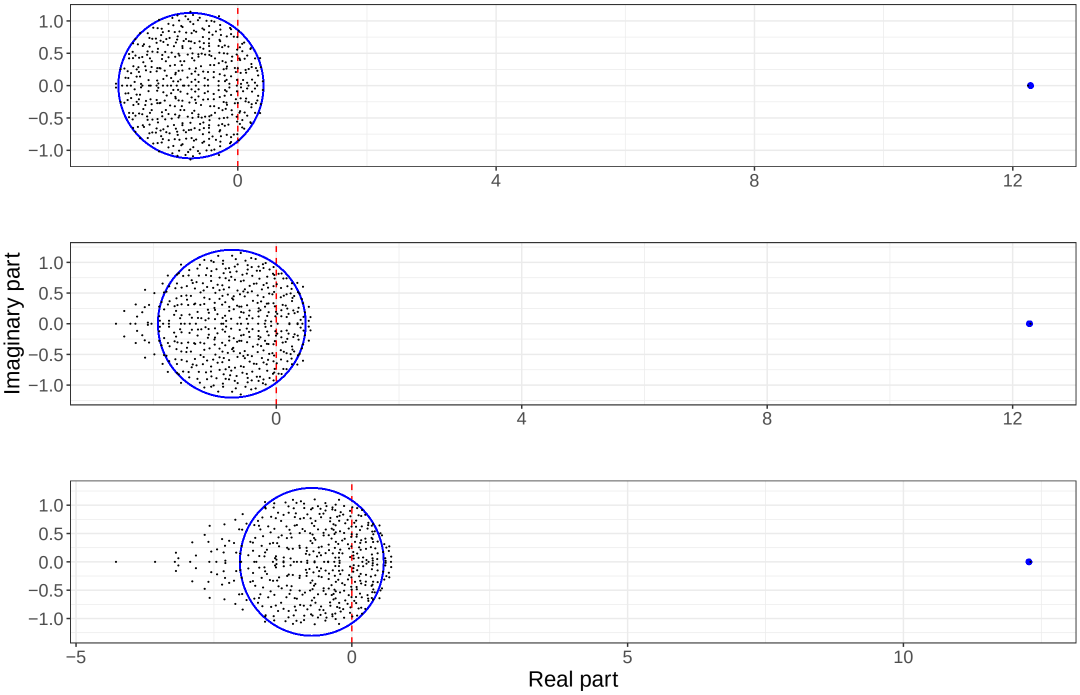

Figure 5: **Variance of the diagonal entries of random matrices.** The convergence of the distribution of the bulk of the eigenvalues to the circular law breaks as σ_m increases. From top to bottom, the migration rates of the corresponding systems have standard deviation $\sigma_m = 0.005, 0.02, 0.03$. All three plots correspond to a Jacobian matrix of size $N = 200$ with off-diagonal entries having mean $\beta\mu_c + \mu_m = 0.026$. The standard deviation of the commuting coefficients is given by $\sigma_c = 0.03$. Note that convergence would not be recovered at higher values of N, as the variance of the diagonal entries in the matrix grows asymptotically with N. In all cases, the outlier eigenvalue matches closely its expected location.

the circle where the bulk of the eigenvalues is contained. Therefore, the stability of the system is determined by a single condition (the location of the outlier eigenvalue), which provides the desired epidemic threshold (Eq. (2) in main text)

$$s(J) = \beta\mu_c(N-1) + \beta - \gamma. \tag{11}$$

1.2 Next-generation matrix

The basic reproduction number R_0 defines the number of contagions caused by a typical infected individual in a fully susceptible population. In compartmental models, it is given by the spectral radius of the next-generation matrix [9, 28]. In this section, we show that RMT could be used to derive R_0 and $s(J)$ for a simplified version of Eq. (1). Both numbers give equivalent epidemic thresholds; this is proved in [10] from the following equality

$$\text{sign}(R_0 - 1) = \text{sign}(s(J)). \tag{12}$$

The equivalence between the two epidemic thresholds follows from Eq. (12):

$$\begin{cases} s(J) < 0 \iff R_0 < 1 : \text{the disease will die out eventually (stability)}, \\ s(J) > 0 \iff R_0 > 1 : \text{the disease may spread over the system (instability)}. \end{cases} \tag{13}$$

Comparing both conditions, we see that $R_0 < 1$ holds if all the eigenvalues of the next-generation matrix are contained in the unit circle (Fig. 6), while $s(J) < 0$ if the maximum real part of the eigenvalues is smaller than zero.

We consider a simplified model with $m_{ij} = 0$ in Eq. (1) and same transmission and recovery rates at the patches, given by

$$\frac{dI_i}{dt} = \beta\frac{S_i}{N_i}I_i - \gamma I_i + \sum_{j=1}^{N}\beta c_{ji}\frac{S_i}{N_i}I_j, \quad i \in \{1, ..., N\}, \tag{14}$$

and show that we can compute $s(J)$ and R_0 with RMT, as well as its equivalence as presented in (13). In order to compute R_0, we follow the algorithm presented in [10] for compartmental models, where R_0 is given by the spectral radius, $\rho(K)$ of the next-generation matrix K. The next-generation matrix is computed as $K = -T\Sigma^{-1}$, where T and Σ can be extracted from the Jacobian matrix as $J = T + \Sigma$. Here, T is the transmission matrix, containing the terms related to production of new infected individuals, and Σ is the transition matrix, containing information relative to changes between infectious states. This decomposition must be chosen in such a way that T is a positive matrix and Σ is a positive off-diagonal matrix with negative dominant eigenvalue. For the infected subsystem (14) we can write

$$T = \begin{pmatrix} \beta & \beta c_{21} & \beta c_{31} & \dots & \beta c_{N1} \\ \beta c_{12} & \beta & \beta c_{32} & \dots & \beta c_{N2} \\ \beta c_{13} & \beta c_{23} & \beta & \dots & \beta c_{N3} \\ \\ \beta c_{1N} & \beta c_{2N} & \beta c_{3N} & \dots & \beta \end{pmatrix}$$

and $\Sigma = \text{diag}(-\gamma, ..., -\gamma)$. The next-generation matrix is then given by

$$K = \frac{\beta}{\gamma}\left(\mu_c \mathbb{1}_N + \mathcal{G}_N(0, \sigma_c) + (1-\mu_c) I_N\right). \tag{15}$$

Following the same reasoning as in section 1.1, we find from RMT results that the eigenvalues of K are uniformly distributed in a circle with radius $(\beta/\gamma)\sigma_c\sqrt{N}$ and center $(\beta/\gamma)(1-\mu_c)$, with an outlier produced by the low-rank matrix $\mu_c \mathbb{1}_N$ given by $(\beta/\gamma)(\mu_c(N-1)+1)$. Therefore, constraining all these eigenvalues to lie inside the unit circle, we find that the basic reproduction number is given by

$$R_0 = \frac{\beta}{\gamma}(\mu_c(N-1)+1) \tag{16}$$

If we compute the epidemic threshold $s(J)$ from the Jacobian matrix,

$$J = \beta\left(\mu_c \mathbb{1}_N + \mathcal{G}_N(0, \sigma_c) + (1-\mu_c) I_N\right) - \gamma I_N, \tag{17}$$

we obtain the following

$$s(J) = \beta(\mu_c(N-1)+1) - \gamma. \tag{18}$$

Equations (16) and (18) present R_0 and $s(J)$, computed using the low-rank perturbation theorem [21] from RMT. Replacing these expressions in the inequalities (13) for the epidemic threshold and the basic reproduction number we see that

$$R_0 < 1 \iff \beta(\mu_c(N-1)+1) < \gamma \iff s(J) < 0. \tag{19}$$

An analogous reasoning holds for the case when $R_0 > 0$, showing the equivalence between both epidemic thresholds $s(J)$ and R_0.

1.3 Gershgorin and Perron-Frobenius theorems

We outline in this section how the classical Gershgorin [29] and Perron-Frobenius [19] theorems may be exploited in the analysis of our model. The less precise estimates provided by these results motivate further our use of RMT techniques.

Let us first consider a model with no short-term mobility between patches. That is, we set all the commuting coefficients c_{ij} to zero in (1), so that the infected subsystem simplifies to

$$\frac{dI_i}{dt} = \beta_i \frac{S_i}{N_i} I_i - \gamma_i I_i - \sum_{j=1}^{N} m_{ji} I_i + \sum_{j=1}^{N} m_{ij} I_j, \qquad i \in \{1, ..., N\}. \tag{20}$$

Around the DFE, the Jacobian of this system of equations reads

$$J = \begin{pmatrix} \beta_1 - \gamma_1 - \sum_j m_{j1} & m_{12} & m_{13} & \dots & m_{1N} \\ m_{21} & \beta_2 - \gamma_2 - \sum_j m_{j2} & m_{23} & \dots & m_{2N} \\ m_{31} & m_{32} & \beta_3 - \gamma_3 - \sum_j m_{j3} & \dots & m_{3N} \\ \vdots & \vdots & \vdots & & \vdots \\ m_{N1} & m_{N2} & m_{N3} & \dots & \beta_N - \gamma_N - \sum_j m_{jN} \end{pmatrix}. \tag{21}$$

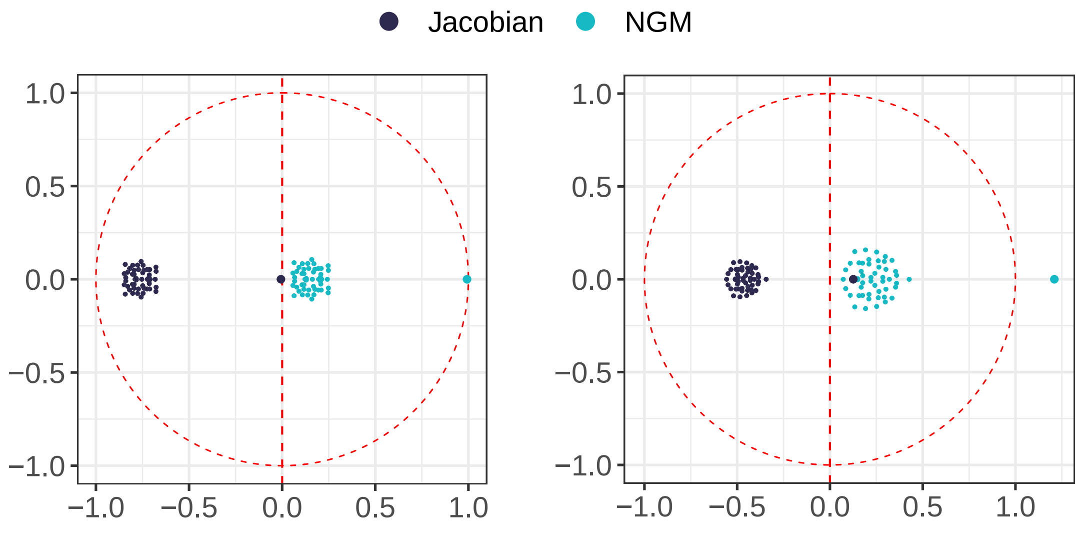

Figure 6: **Eigenvalue distribution for the Jacobian and the next-generation matrix.** Eigenvalue distribution for the Jacobian J and the next-generation matrix K of a stable system (left). The red dashed lines show the stability boundaries for the epidemic threshold $s(J)$ (vertical line) and the reproduction number R_0 (unit circle). Eigenvalue distribution for an unstable system (right). The Jacobian has one eigenvalue with real part greater than zero. Accordingly, one eigenvalue of the next-generation matrix of the system is larger than 1 in modulus.

Using Gershgorin's circle theorem on the columns of this matrix, we see that its eigenvalues are contained in the union of the disks

$$D\left(\beta_i - \gamma_i - \sum_j m_{ji}, \sum_j m_{ji}\right),$$

for $i \in \{1, \ldots, N\}$. Therefore, a sufficient condition for the stability of the system is that the transmission rates β_i are smaller than the exit rates γ_i at all patches i, regardless of the values taken by the mobility coefficients m_{ji}. Indeed, this is a consequence of the fact that infected individuals only move between patches in this case, without generating new infections in the process. Therefore, the only quantities that influence the growth of the disease are the rates β_i and γ_i at which new infections are being generated and depleted at each patch. An analogous phenomenon has been observed in [25] in the context of plant epidemics, see also [16] for a different proof of this fact. Thus, the stability of the system is determined by the local growth rates $\beta_i - \gamma_i$ at the nodes of the network.

We can also consider a system with nonzero commuting flows and bound the dominant eigenvalue of the Jacobian matrix using Perron-Frobenius theorem. For the simplest case of constant transmission and depletion rates across nodes, the theorem provides the bounds

$$\min_j \sum_i J_{ij} = \beta - \gamma + \beta \min_i \sum_i c_{ji} \leq s(J) \leq \max_j \sum_i J_{ij} = \beta - \gamma + \beta \max_i \sum_i c_{ji}, \qquad (22)$$

in terms of the entries J_{ij} of the Jacobian matrix given in Eq. (3). These locate the epidemic threshold in an interval containing the point $\beta - \gamma + \beta\mu_c(N-1)$, with the width of the interval depending on the variance of the commuting rates σ_c. Already in this case the RMT techniques provide a better estimate, providing an exact expression for $s(J)$. A more significant improvement is found for the several generalizations of the model described in the following sections below: while RMT techniques provide in all cases an exact, analytic expression for $s(J)$, the bounds obtained from Gershgorin and Perron-Frobenius theorems become much less precise.

2 Transmission and disease distribution

In Section 1.1 we obtained the epidemic threshold for a system with the same transmission and recovery rates at all patches. In this section we relax this assumption and study more general scenarios. Let us assume that at k nodes of the network, the transmission rate is $\beta^* = \beta + \alpha$. This could model the effect of successful vaccination, efficient public health measures ($\alpha < 0$) or massive gatherings ($\alpha > 0$), for instance. Labelling these nodes as the first k ones, without loss of generality, the Jacobian of the system around the DFE equals

$$\begin{pmatrix} \beta+\alpha-\gamma-\sum_j m_{j1} & \dots & (\beta+\alpha)c_{k1}+m_{1k} & \beta c_{(k+1)1}+m_{1(k+1)} & \dots & \beta c_{N1}+m_{1N} \\ \vdots & \ddots & \vdots & \vdots & & \vdots \\ (\beta+\alpha)c_{1k}+m_{k1} & \dots & \beta+\alpha-\gamma-\sum_j m_{jk} & \beta c_{(k+1)k}+m_{k(k+1)} & \dots & \beta c_{Nk}+m_{kN} \\ (\beta+\alpha)c_{1(k+1)}+m_{(k+1)1} & \dots & (\beta+\alpha)c_{k(k+1)}+m_{(k+1)k} & \beta-\gamma-\sum_j m_{j(k+1)} & \dots & \beta c_{N(k+1)}+m_{(k+1)N} \\ \vdots & & \vdots & \vdots & \ddots & \vdots \\ (\beta+\alpha)c_{1N}+m_{N1} & \dots & (\beta+\alpha)c_{kN}+m_{Nk} & \beta c_{(k+1)N}+m_{N(k+1)} & \dots & \beta-\gamma-\sum_j m_{jN} \end{pmatrix}.$$

Using the same reasoning as in section 1.1, the eigenvalue distribution of this matrix can be seen to coincide with that of matrix (4) upon replacing the structure matrix by

$$\begin{pmatrix} \beta\mu_c+\alpha+\mu_m & (\beta+\alpha)\mu_c+\mu_m & \dots & (\beta+\alpha)\mu_c+\mu_m & \beta\mu_c+\mu_m & \dots & \beta\mu_c+\mu_m \\ (\beta+\alpha)\mu_c+\mu_m & \beta\mu_c+\alpha+\mu_m & & (\beta+\alpha)\mu_c+\mu_m & \beta\mu_c+\mu_m & \dots & \beta\mu_c+\mu_m \\ \vdots & \vdots & \ddots & \vdots & \vdots & & \vdots \\ (\beta+\alpha)\mu_c+\mu_m & (\beta+\alpha)\mu_c+\mu_m & \dots & \beta\mu_c+\alpha+\mu_m & \beta\mu_c+\mu_m & \dots & \beta\mu_c+\mu_m \\ (\beta+\alpha)\mu_c+\mu_m & (\beta+\alpha)\mu_c+\mu_m & \dots & (\beta+\alpha)\mu_c+\mu_m & \beta\mu_c+\mu_m & \dots & \beta\mu_c+\mu_m \\ \vdots & \vdots & & \vdots & \vdots & \ddots & \vdots \\ (\beta+\alpha)\mu_c+\mu_m & (\beta+\alpha)\mu_c+\mu_m & \dots & (\beta+\alpha)\mu_c+\mu_m & \beta\mu_c+\mu_m & \dots & \beta\mu_c+\mu_m \end{pmatrix}, \quad (23)$$

where a total of k columns have been modified in (23). We assume that the number of perturbed nodes k is small compared to N, to ensure that the low-rank perturbation theorem [21] provides a factual estimate for the corresponding eigenvalue distribution. Now k outliers arise in the eigenvalue distribution of the Jacobian, stemming from the k nontrivial eigenvalues of the matrix (23), with the largest one being equal to (Eq. (9) in main text)

$$\begin{aligned} s(J) = {} & \frac{N}{2}\left(\beta\mu_c+\mu_m\right)+\frac{\alpha}{2}\left(1+(k-1)\mu_c\right)+\beta(1-\mu_c)-\gamma-N\mu_m \qquad (24) \\ & +\frac{1}{2}\sqrt{N^2\left(\beta\mu_c+\mu_m\right)^2+\alpha^2\left(1+(k-1)\mu_c\right)^2+2\alpha\left(\beta\mu_c+\mu_m\right)\left[N\left(1+(k-1)\mu_c\right)+2(N-k)\left(\mu_c-1\right)\right]}. \end{aligned}$$

The resulting expression for the epidemic threshold is more complicated than that for the system with equal transmission rates. We also find a dependence on the average migratory flow μ_m, in addition to the expected dependence on the perturbation in transmission α and the number of affected nodes k (see Fig. 7 for examples). Interestingly, an increase in transmission in one patch to $\beta^* = \beta + k\alpha$ produces a stronger variation in the stability than an equivalent increase in k patches to $\beta^* = \beta + \alpha$ (Fig.8b). We also find that Eq. (24) gives a better prediction for the epidemic threshold than the one obtained from the circular law Eq. (11) when considering the average transmission rate of the perturbed system $\beta' = (k\beta^* + (N-k)\beta)/N$ (Fig.8a).

In addition to this, we may also consider random fluctuations in the transmission rates across patches, drawing β_i from a probability distribution with mean μ_β and variance σ_β. Numerical experiments show that the eigenvalue distribution for the system with random β_i is remarkably close to that predicted with a fixed diagonal term, corresponding to the mean of the random vector, μ_β. The epidemic threshold would thus read as follows

$$s(J) = \mu_\beta\mu_c(N-1) + \mu_\beta - \gamma. \qquad (25)$$

This prediction seems to be less accurate as either the coefficient of variation or the variance of the distribution followed by the β_i increases (Fig. 9). Larger values of N provide a better agreement, and the larger the variance of the off-diagonal terms of J is with respect to the variance of its diagonal terms, the better the predicted distribution matches the empirically found one. This could model the case where there are some patches with zero or close to zero transmission rate. Even in this case, the average μ_β determines the stability of the system and the number of infected

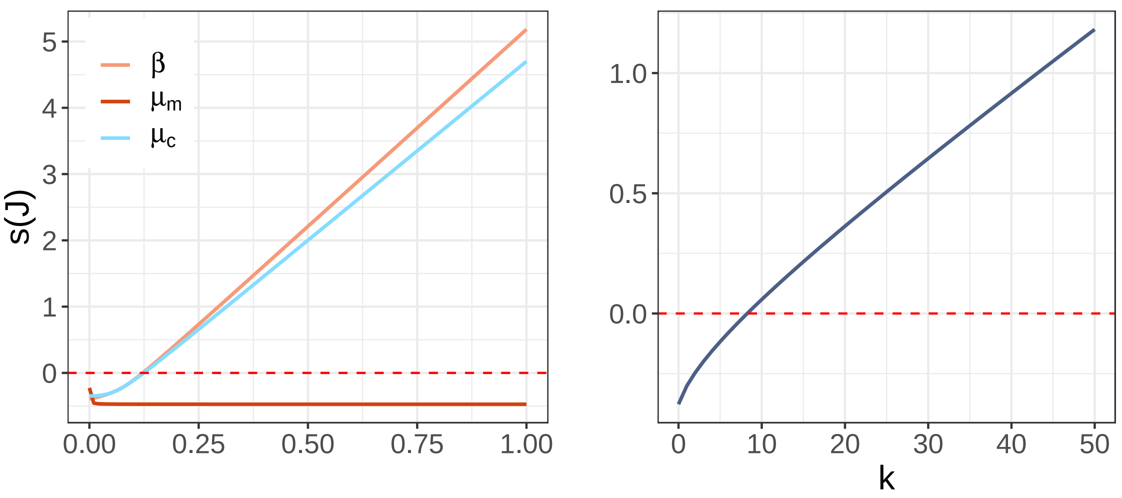

Figure 7: **Epidemic threshold $s(J)$ for systems with perturbed transmission.** Changes in Eq. (24) upon variations in several parameters of a system with $N = 100$, $\beta = 0.05$, $\gamma = 0.8$, $\mu_c = 0.05$, $\mu_m = 0.001$. As commuting and transmission increase (left plot, for a system with $k = 1$ modified patch with transmission $\beta + \alpha = 0.55$) the rightmost eigenvalue increases. We observe that a relatively smaller variation in the epidemic threshold is caused by μ_m, consistently with the unperturbed setting. The right plot shows the influence of the number of patches where the transmission is higher. As expected, as the number of modified patches increases the rightmost eigenvalue increases.

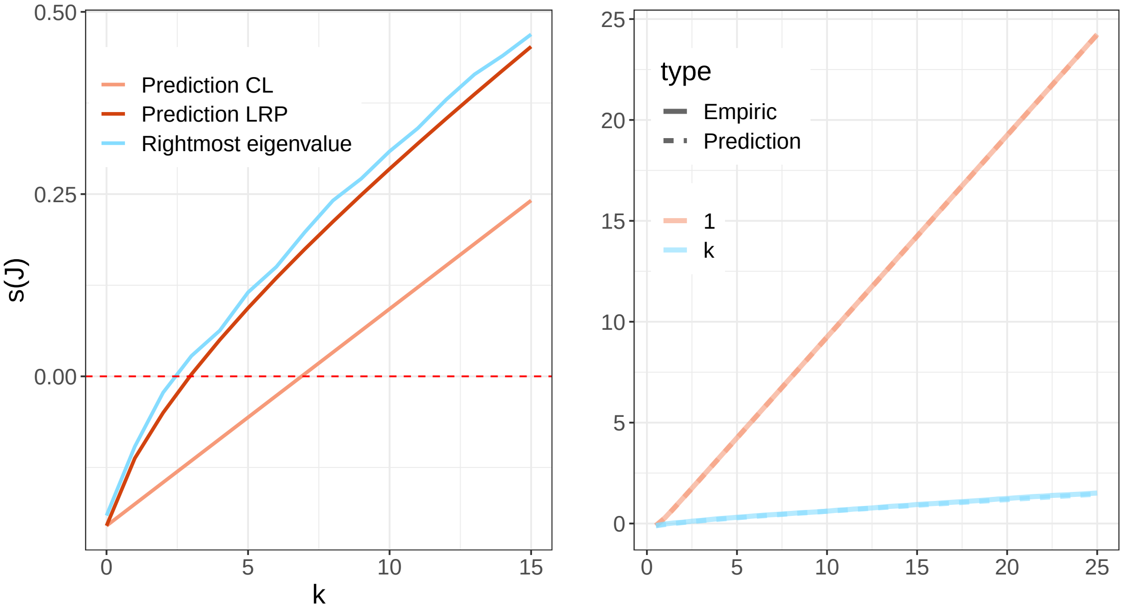

Figure 8: **Variations in transmission at several patches of the system.** Accuracy of the low-rank perturbation theorem (left). A better estimate is obtained for the rightmost eigenvalue in comparison to that obtained with Eq. (11) by simply using the average transmission rate of the perturbed system (size $N = 100$). Comparison between the epidemic threshold after increasing the transmission rate in a single patch to $\beta^* = \beta + k\alpha$ versus increasing it at k patches to $\beta^* = \beta + \alpha$ (right), for $\beta = 0.05$ and $\alpha = 0.5$. There is a greater change in the rightmost eigenvalue when the increase is localized at one node compared to k nodes.

individuals may grow at all the patches. This is consistent with the results in [11, 25], where it is proved that if the network is fully connected then almost surely there is no equilibrium point having patches with no infected individuals.

In section 1.1, the recovery rates were also assumed to be constant. We can also relax this assumption and assume that the recovery rate γ_i in each patch is drawn from a probability distribution with mean μ_γ and variance σ_γ. This results in the diagonal entries of the Jacobian matrix having finite variance, which is still under the assumptions of the low-rank perturbation theorem [21]. We therefore obtain the same epidemic threshold in Eq. (11) in this case, upon replacing γ by μ_γ.

Let us note that two seemingly contradicting phenomena have been described in this section. First, a different epidemic threshold has been obtained for targeted perturbations in the transmission rates of the system at a small number of patches by means of the low-rank perturbation theorem. In particular, this expression caused a larger increase in $s(J)$ whenever an equivalent rise in transmission was concentrated at fewer locations. Secondly, we have observed a very close agreement between systems with random variations in their transmission rates and those with equal rates across patches. These findings seem to be incompatible, as the concentrated and disseminated increases in transmission considered in the first case were equal in mean. This fact may be understood in terms of the different nature of the two perturbations of the system. The first change favours a more severe rupture of the otherwise statistically balanced distribution of the underlying random matrix, while the second generates smoother statistical fluctuations that are compensated more easily. This is a subtle distinction that will arise also when considering variations on the mobility networks.

3 Random mobility networks

The epidemic threshold (Eq. (11)) shows that the average commuting flow is the only parameter from the connectivity networks that may influence the possibility of spread of the disease on unperturbed systems. Other features of the mobility flows may cause qualitative changes in the evolution of the pandemic, relevant for adequate design of public health measures. As explained in the main article, networks with a more heterogeneous distribution of commuting and migration flows result in more heterogeneous epidemic landscapes, in which patches with both higher and lower incidences can be found, see Fig. 10.

An instance of a more heterogeneous setting results from considering sparse connectivity networks. The examples of system (Eq. (1)) considered until now displayed fully connected networks. Here, we explore the stability of the system with a sparse connectivity network, i.e. a proportion of the flows is set to zero. In order to do this, each of the coefficients in the connectivity matrix is multiplied by a Bernoulli random variable with mean p; this models the effect of removing a proportion p of the connectivity flows of the system. This scenario has the same epidemic threshold as a system where the mean of the migration and the commuting coefficients is multiplied by the probability of success of the Bernoulli variable, $\mu = p(\beta\mu_c + \mu_m)$. The latter represents a uniform

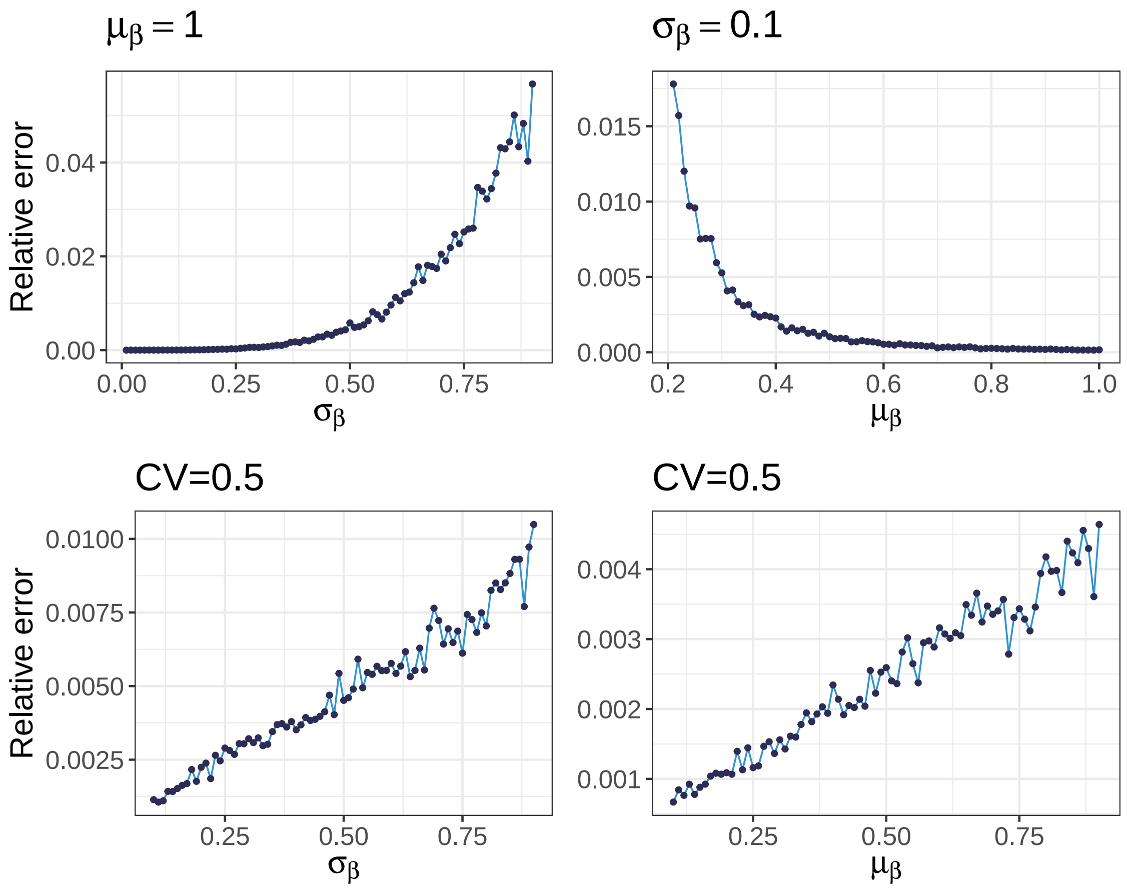

Figure 9: **Accuracy on predictions for random transmission rates** β_i**.** Relative error for the outlier eigenvalue predicted by Eq. (11) for a system of $N = 200$ nodes with random transmission rates. Top row shows relative error in terms of the standard deviation σ_β and a fixed average $\mu_\beta = 1$ (top left) and in terms of the average μ_β with a fixed standard deviation σ_β (top right). Bottom row shows relative error for the system in terms of the standard deviation σ_β (bottom left) and the average μ_β (bottom right) of the transmission rates for a fixed coefficient of variation $CV = \sigma_\beta/\mu_\beta = 0.5$. If we compare the different scenarios, we observe a linear increase in loss of accuracy whenever CV is constant for either σ_β and μ_β. On the other hand, the relative accuracy of the prediction when CV is not fixed seems to depend non-linearly on σ_β and μ_β (and thus on CV as well). We see that as the CV increases the accuracy of the prediction decreases.

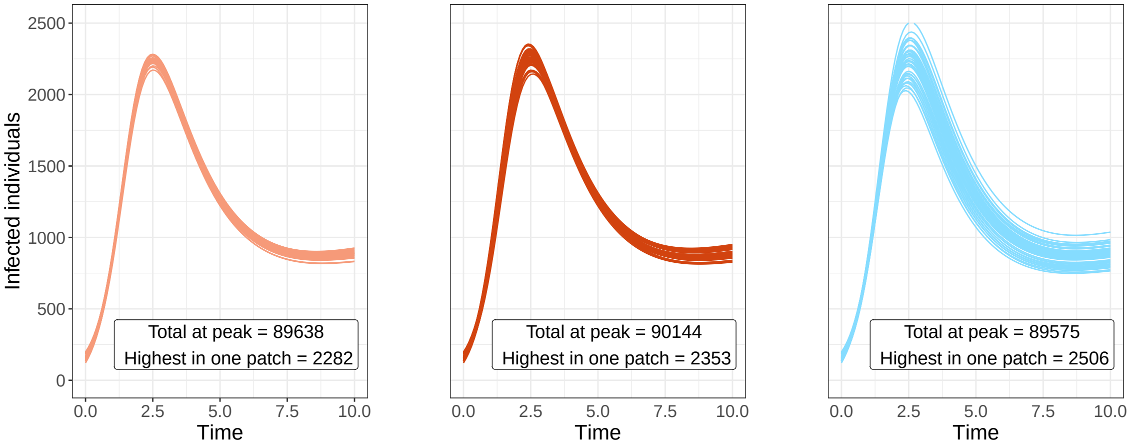

Figure 10: **Variability in the connectivity networks and epidemic heterogeneity.** Increasing either the networks' joint variance (center, from $\sigma = 0.01$ to $\sigma = 0.03$) or the average migration flow (right, from $\mu_m = 0.001$ to $\mu_m = 0.01$) increases the heterogeneity in disease incidence across patches with respect to the base scenario (left, $N = 40$).

proportional reduction in the strength of the connectivity on all of the flows of the system. Since the outlier eigenvalues coincide for both systems, we find the same qualitative behaviour for the spread of the disease (Fig. 11). However, the connectivity matrices are distinct and thus the bulk of the eigenvalues changes its shape: the radius of the circle differs due to the increase in variance in the sparse network (Fig. 11). In particular, the larger variance of the sparse system results in a more heterogeneous distribution of the incidence across patches.

3.1 Restrictions in commuting flows

We have observed the influence of the average commuting flow μ_c in the epidemic threshold (Eq. (11)) when this remains the same for all the coefficients in the network. Let us explore the influence in the epidemic threshold of structured modifications in the commuting network. For this, we follow the same reasoning as in section 2 for the case of variable transmission rates. More precisely, we replace the structure matrix in (4) by one that adequately models a given modified mobility network, and compute the corresponding outlier by means of the low-rank perturbation theorem [21]. We consider a perturbation $\mu_c \to \mu_c + \nu$ on the average commuting flow on some flows of the network, which increases or decreases the strength of the connectivity depending on the sign of ν, and assume that a number k of patches or a proportionate number of connectivity flows are perturbed. As above, we assume k to be small compared to the size of the network N in order for the low-rank perturbation theorem [21] to hold.

We first consider targeted scenarios, where there is a set of k patches with modified average commuting flows: these comprise all the outgoing (A), incoming (B) or incoming and outgoing (C) flows. In this case, rows and/or columns in the structure matrix are modified. We also

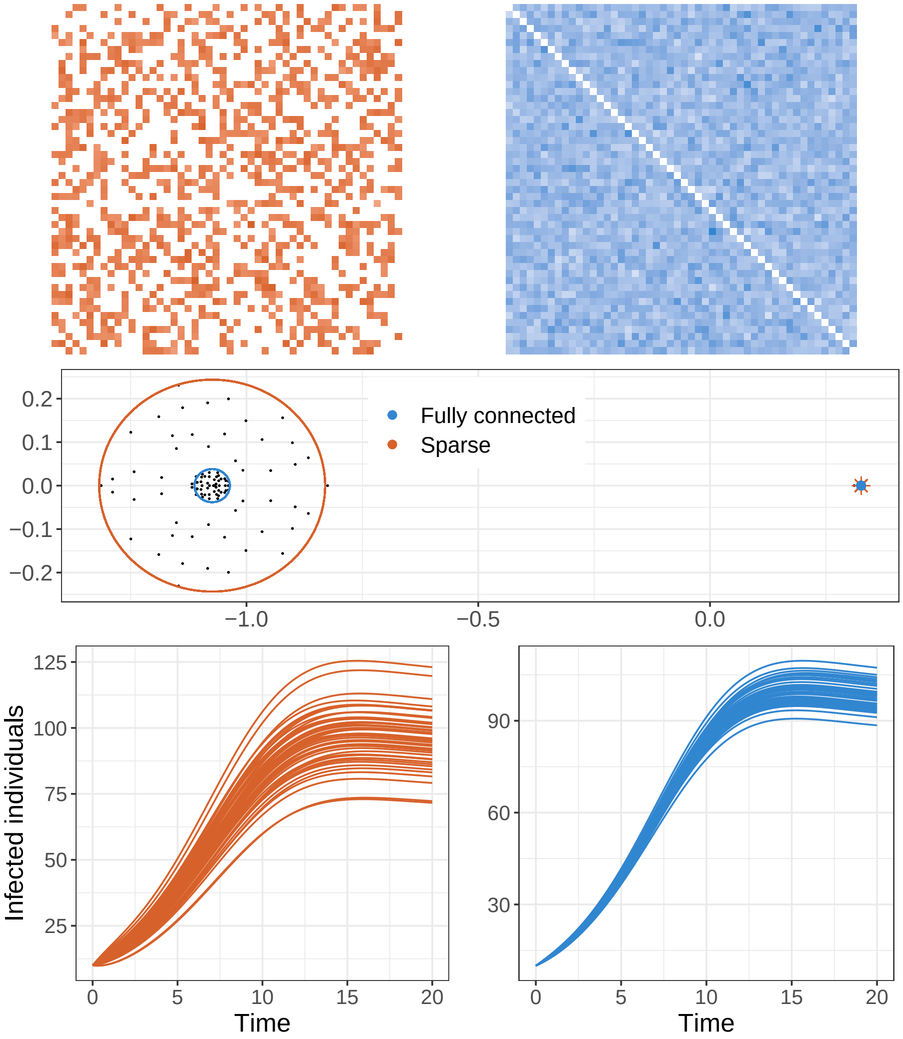

Figure 11: **Sparse connectivity networks.** Mobility network (top), eigenvalue distribution (center) and evolution of the number of infected individuals in time (bottom) for a sparse system (left, orange) and a uniformly restricted scenario (right, blue), with $N = 50$ and a proportion $p = 0.6$ of flows set to zero. Accordingly, the fully connected system shows a 60% reduction in the connectivity flows. The outliers are in the same position, in both scenarios the number of infected individuals increases initially. The bulk of eigenvalues is different, with higher variance for the sparse matrix implying a larger radius for the bulk of the eigenvalues and thus a higher variability between nodes.

study non-structured scenarios: varying randomly chosen coefficients in the connectivity matrix (D), introducing symmetry in this random choice by modifying flows in both directions (E), and reducing uniformly the average commuting flow throughout the network (F). These scenarios are modelled as follows:

- **Strategy A: outgoing flows at selected locations**. The structure matrix in (4) is now

$$\begin{pmatrix} \beta\mu_c+\mu_m & \beta(\mu_c+\nu)+\mu_m & \dots & \beta(\mu_c+\nu)+\mu_m & \beta\mu_c+\mu_m & \dots & \beta\mu_c+\mu_m \\ \beta(\mu_c+\nu)+\mu_m & \beta\mu_c+\mu_m & & \beta(\mu_c+\nu)+\mu_m & \beta\mu_c+\mu_m & \dots & \beta\mu_c+\mu_m \\ \vdots & \vdots & \ddots & \vdots & \vdots & & \vdots \\ \beta(\mu_c+\nu)+\mu_m & \beta(\mu_c+\nu)+\mu_m & \dots & \beta\mu_c+\mu_m & \beta\mu_c+\mu_m & \dots & \beta\mu_c+\mu_m \\ \vdots & \vdots & & \vdots & \vdots & \ddots & \vdots \\ \beta(\mu_c+\nu)+\mu_m & \beta(\mu_c+\nu)+\mu_m & \dots & \beta(\mu_c+\nu)+\mu_m & \beta\mu_c+\mu_m & \dots & \beta\mu_c+\mu_m \end{pmatrix}, \tag{26}$$

  where the first k columns of the matrix have been modified, and the last $(N-k)$ remain unchanged. For this choice of structure matrix, the outlier of the Jacobian (3) is given by

$$\begin{aligned} s(J) = {} & \frac{N}{2}\left(\beta\mu_c+\mu_m\right)+\frac{k-1}{2}\beta\nu+\beta\left(1-\mu_c\right)-\gamma-N\mu_m \\ & +\frac{1}{2}\sqrt{N^2\left(\beta\mu_c+\mu_m\right)^2+(k-1)^2\beta^2\nu^2+2(N+Nk-4k)\left(\beta\mu_c+\mu_m\right)\beta\nu}. \end{aligned} \tag{27}$$

- **Strategy B: incoming flows at selected locations**. The structure matrix is precisely the transpose of the structure matrix for strategy A, and thus the corresponding epidemic threshold coincides with Eq. (27).

- **Strategy C: incoming and outgoing flows at selected locations**. The structure matrix in (4) is now

$$\begin{pmatrix} \beta\mu_c+\mu_m & \dots & \beta(\mu_c+\nu)+\mu_m & \beta(\mu_c+\nu)+\mu_m & \dots & \beta(\mu_c+\nu)+\mu_m \\ \beta(\mu_c+\nu)+\mu_m & \dots & \beta(\mu_c+\nu)+\mu_m & \beta(\mu_c+\nu)+\mu_m & \dots & \beta(\mu_c+\nu)+\mu_m \\ \vdots & \ddots & \vdots & \vdots & & \vdots \\ \beta(\mu_c+\nu)+\mu_m & \dots & \beta\mu_c+\mu_m & \beta(\mu_c+\nu)+\mu_m & \dots & \beta(\mu_c+\nu)+\mu_m \\ \beta(\mu_c+\nu)+\mu_m & \dots & \beta(\mu_c+\nu)+\mu_m & \beta\mu_c+\mu_m & \dots & \beta\mu_c+\mu_m \\ \vdots & & \vdots & \vdots & \ddots & \vdots \\ \beta(\mu_c+\nu)+\mu_m & \dots & \beta(\mu_c+\nu)+\mu_m & \beta\mu_c+\mu_m & \dots & \beta\mu_c+\mu_m \end{pmatrix}, \tag{28}$$

  where the first k rows and columns of the matrix have been modified and bottom-right $(N-k)\times(N-k)$ block remains unchanged. For this choice of structure matrix, the outlier of the Jacobian (3) is given by

$$\begin{aligned} s(J) = {} & \frac{N}{2}\left(\beta\mu_c+\mu_m\right)+\frac{k-1}{2}\beta\nu+\beta\left(1-\mu_c\right)-\gamma-N\mu_m \\ & +\frac{1}{2}\sqrt{N^2\left(\beta\mu_c+\mu_m\right)^2+2\beta\nu(\beta\mu_c+\mu_m)(N+k(3N-2k-2))+\beta^2\nu^2\left(4k(N-k)+(k-1)^2\right)}. \end{aligned} \tag{29}$$

- **Strategy D, E and F: randomly chosen flows**. The structure matrix is now replaced by $(\beta(\mu_c + \nu k/N) + \mu_m)\mathbb{1}_N$. The location of the outlier is now provided by the same expression as in the unperturbed case (3), with the corresponding modified average commuting flow

$$s(J) = (N-1)\beta(\mu_c + \nu k/N) + \beta - \gamma. \tag{30}$$

  As explained above when considering sparse matrices, the average commuting flow for strategy D and F coincide if the number of perturbed flows is the same, and thus the resulting epidemic thresholds are also equal. Strategy E consists of modifying the mobility flows in both directions at half as many randomly chosen edges of the network. While this perturbation results in a non-zero correlation between the diagonally opposite elements of the random matrix, this is known to only modify the distribution of the bulk of the eigenvalues [21], and thus results in the same epidemic threshold as strategy D as well. See section 3.3 below for more complicated correlations in the mobility matrices and their effect on the spread of the disease.

Fig. 12 shows variations in the epidemic threshold $s(J)$ corresponding to strategies A-F above upon variations in several relevant parameters of the system.

3.2 Perturbed migration scenarios

To investigate the response of the system to perturbations in the migration network, we compare in Fig. 13 six scenarios of increased migratory flows analogous to those considered in the previous section. We consider increased incoming flows to particular patches as well as outgoing flows, and non-structured modifications of the network. These could represent scenarios of humanitarian crisis or seasonal movements between particular locations, for instance.

As expected, the possibility of spread of the disease remains the same upon these perturbations in the network (see Results in main article and section 1 above). However, sensible differences arise in the local distribution of infected individuals over the system. For Scenario A (B), that models increased incoming (outgoing) migratory flows at certain nodes, we find that a larger (smaller) number of infected individuals concentrate at these locations, as a consequence of the overall larger number of individuals moving to (out of) the patches. If both incoming and outgoing flows are increased (Scenario C), these compensate each other and no significant differences arise in the affected patches. Among the randomly perturbed scenarios, those introducing more variability in the network (D and F) result in more heterogeneous epidemic landscape across patches.

3.3 Generally correlated networks

Baron *et al* [6] provide an asymptotic expression for the eigenvalue distribution of a random matrix with general pair-to-pair correlations between their elements. These may modify the location of both the bulk and the outlier eigenvalues, and thus change the stability of the corresponding system. Let us review their result and its implications for our model.

Consider a random matrix which elements z_{ij} are drawn from a probability distribution with mean μ/N and variance σ^2/N. Following [6], we assume that elements in the random matrix may

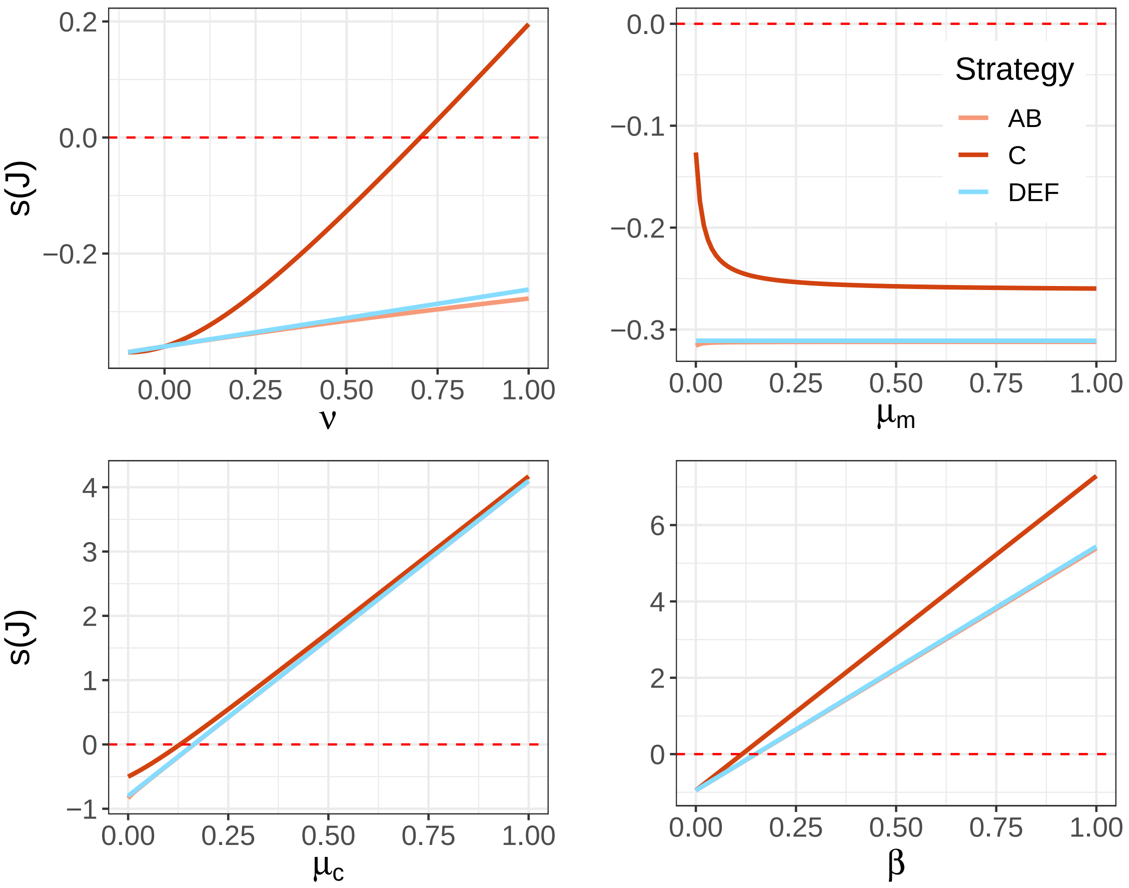

Figure 12: **Epidemic threshold $s(J)$ for systems with perturbed commuting networks.** Changes in the epidemic thresholds Eqs. (27),(29),(30) upon variations in several system parameters (from top-left to bottom-right): perturbation factor ν, average migration flow μ_m, average commuting flow μ_c, transmission rate β. For the base scenario the parameters are $N = 50$, $\beta = 0.1$, $\gamma = 0.95$, $\mu_c = 0.1$, $\mu_m = 0.0001$, $k = 1$ and $\nu = 0.5$. Increases in ν, μ_c, and β increase $s(J)$, being strategy C the most sensitive to these increases, particularly for ν and β. An increase of μ_m (top right) produces a decrease in $s(J)$ for strategy C, while strategies AB and DEF remain close to constant in comparison. This corroborates the smaller influence of the migration flows in the stability of the system observed in Sections 1.1 and 2.

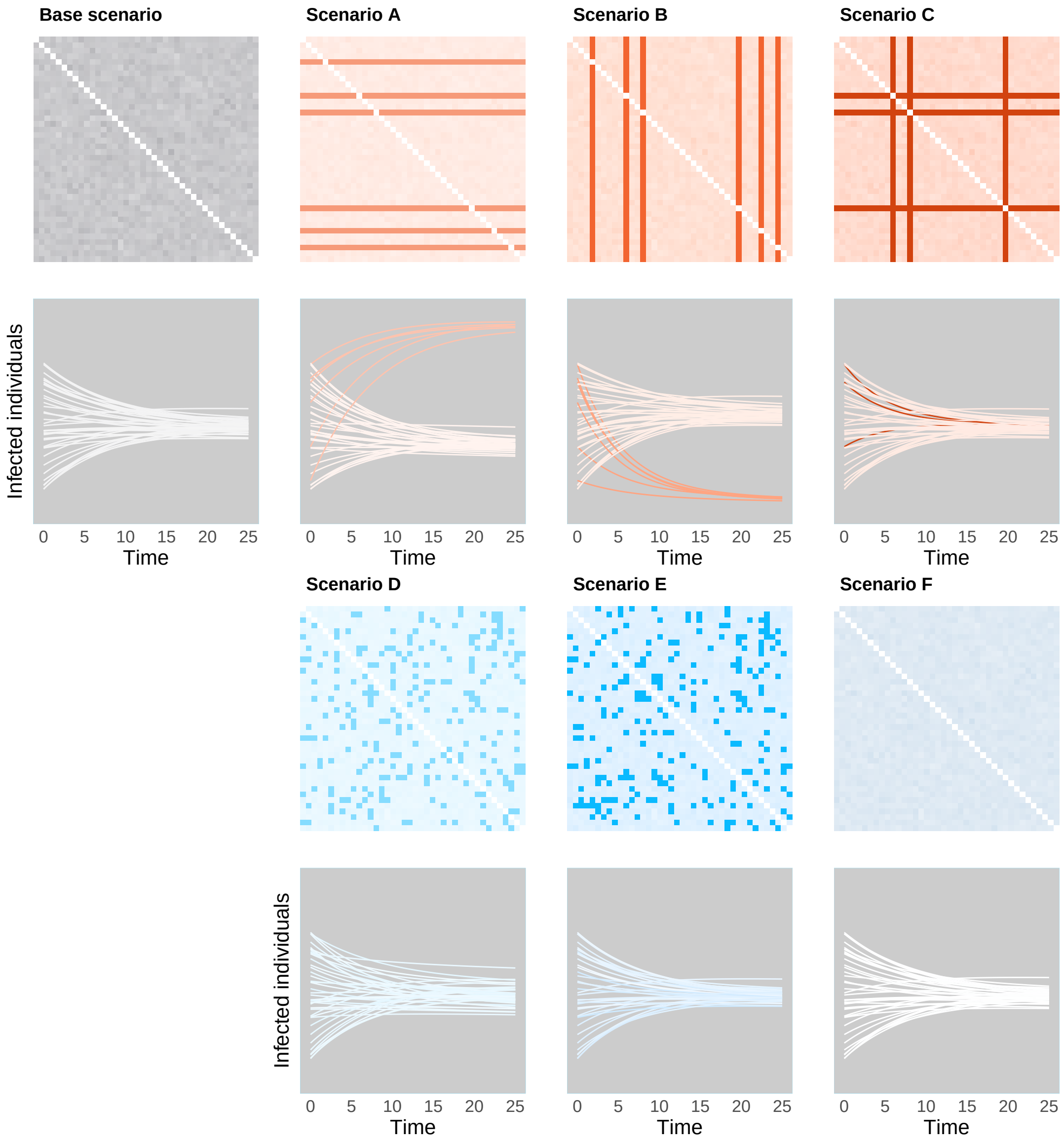

Figure 13: **Variations in the migration network.** We consider a base scenario (size $N = 40$) of sustained disease spread (positive, close to zero value for $s(J)$, in grey) and test six perturbed migratory scenarios analogous to those described for the commuting network in the main article, here for an increase in migratory flows from $\mu_m = 0.0005$ to $\mu_m^* = 0.0025$. Migratory rates concerning a total of 6 nodes or an equivalent proportion of flows are considered in the figure. In each scenario, the top graph displays the strength of the migration flow from patch i to patch j in its (i, j)-th cell. The brightness of the color represents the strength of the flow, with white representing absence of movement. Each line in the bottom graph shows the evolution of the infected population at the patches, colored according to its average incoming and outgoing migratory flows. The three targeted scenarios (in shades of orange) consist of A: increased incoming migration, B: increased outgoing migration, C: increased incoming and outgoing migration at half of the nodes. The three random scenarios (in shades of blue) consist of D: increasing randomly chosen unidirectional flows, E: increasing half as many randomly chosen flows in both directions, F: uniformly increasing all the flows in the network.

also display non-trivial correlations with other matrix coefficients located at specific positions. More precisely, matrix elements that share a common index verify[1]

$$
\begin{aligned}
\rho &= \mathrm{Cor}(z_{ij}, z_{ji}),\\
\frac{\Gamma}{N} &= \mathrm{Cor}(z_{ij}, z_{ki}),\\
\frac{r}{N} &= \mathrm{Cor}(z_{ij}, z_{ik}),\\
\frac{c}{N} &= \mathrm{Cor}(z_{ji}, z_{ki}).
\end{aligned}
\tag{31}
$$

Then, as $N \to \infty$, the bulk of the eigenvalues of the matrix distribute uniformly on the ellipse centered at the origin with semi-axes $(1+\rho)\sigma$ and $(1-\rho)\sigma$, following the well-known elliptic law [20]. There is also a single outlier eigenvalue, located at

$$
\mu + \frac{\mu}{2}\left(1 + \frac{\rho}{\Gamma}\right)\left(\sqrt{1 + \frac{4\Gamma\sigma^2}{\mu^2}} - 1\right). \tag{32}
$$

This coincides with the location given by the low rank perturbation theorem (first term in the sum), with a translation that depends on the value of the correlations ρ and Γ, as well as the mean and variance of the matrix coefficients z_{ij} (second addend). Additionally, Baron *et al* [6] show that any pair-to-pair correlations between elements of the matrix other than those in (31) may be absorbed in the mean of the coefficients z_{ij} in the large-N limit. Therefore, eigenvalue predictions for networks that display more complex inter-dependencies can be approximated with accuracy by the expression (32) as long as N is sufficiently large. Interestingly, we see from this expression that the correlations r and c in (31) do not modify the eigenvalue distribution of the random matrix.

The parameters describing the statistical variability of the Jacobian matrix in our model can be obtained from those of the commuting and migration coefficients. Indeed, the off-diagonal terms of J matrix can be expressed as a linear combination of two differently distributed random matrices

$$
\beta \begin{pmatrix}
0 & c_{21} & c_{31} & \dots & c_{N1}\\
c_{12} & 0 & c_{32} & \dots & c_{N2}\\
c_{13} & c_{23} & 0 & \dots & c_{N3}\\
\vdots & \vdots & \vdots & & \vdots\\
c_{1N} & c_{2N} & c_{3N} & \dots & 0
\end{pmatrix}
+
\begin{pmatrix}
0 & m_{12} & m_{13} & \dots & m_{1N}\\
m_{21} & 0 & m_{23} & \dots & m_{2N}\\
m_{31} & m_{32} & 0 & \dots & m_{3N}\\
\vdots & \vdots & \vdots & & \vdots\\
m_{N1} & m_{2N} & m_{N3} & \dots & 0
\end{pmatrix}. \tag{33}
$$

Let us denote as follows the correlations between matrix elements in both of these matrices (note the different scaling in N with respect to (31))

$$
\begin{aligned}
\rho_c &= \mathrm{Cor}(c_{ij}, c_{ji}), & \rho_m &= \mathrm{Cor}(m_{ij}, m_{ji}),\\
\Gamma_c &= \mathrm{Cor}(c_{ij}, c_{ki}), & \Gamma_m &= \mathrm{Cor}(m_{ij}, m_{ki}),\\
r_c &= \mathrm{Cor}(c_{ij}, c_{ik}), & r_m &= \mathrm{Cor}(m_{ij}, m_{ik}),\\
c_c &= \mathrm{Cor}(c_{ji}, c_{ki}), & c_m &= \mathrm{Cor}(m_{ji}, m_{ki}).
\end{aligned}
\tag{34}
$$

[1]We use the usual notation ρ in place for Baron *et al*'s Γ, and replace their γ by Γ to avoid confusion with our rate of depletion of infected individuals. All indices i, j, k in (31) are assumed distinct.

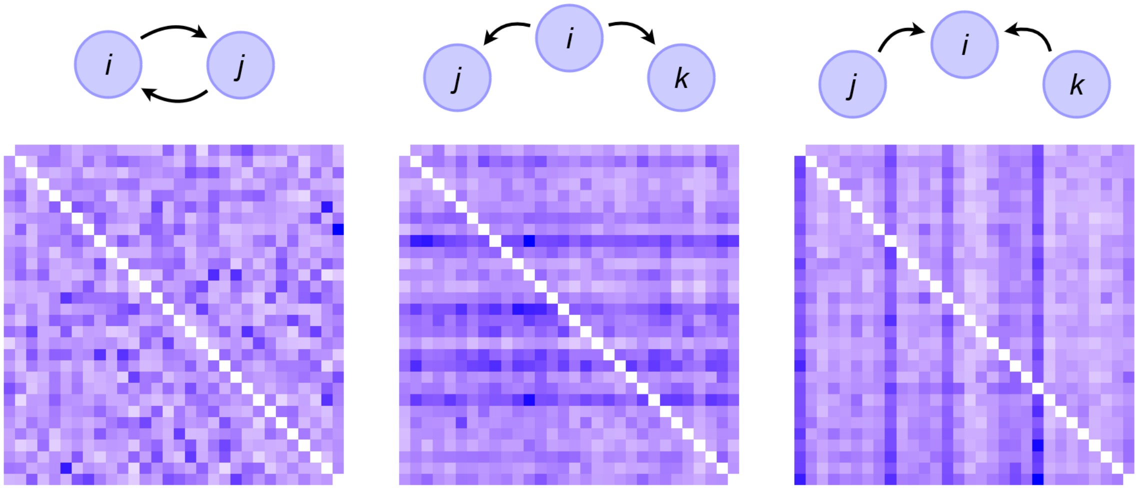

Figure 14: **Correlated mobility networks.** Commuting networks of size $N = 30$ displaying three types of correlation between their flows. Network showing a positive value for ρ ($\rho = 0.85$), see Eq.(34)), that concerns flows between patches going in opposite directions (left), thus favoring a more symmetric configuration with respect to the diagonal. Networks showing positive values for r and c ($r = 0.7$ and $c = 0.7$ respectively, see Eq. (34)), which concern outgoing and incoming flows at the patches (center and right, respectively). These correlations result in more homogeneous distributions across the columns and rows of the mobility matrix respectively, favouring the existence of "source" or "sink" locations. See Fig. 5 in main article for examples of networks displaying nonzero Γ correlation.

See Figure 14 for some examples of correlated networks and the meaning of each parameter in (31) in the context of mobility networks. The relevant parameters encoding the statistical variability of the joint mobility network result from combining those of the two random matrices in (33)

$$\begin{aligned}
\sigma &= \sqrt{\beta^2\sigma_c^2 + 2\beta\tau + \sigma_m^2},\\
\rho &= (\rho_c\beta^2\sigma_c^2 + \rho_m\sigma_m^2)/\sigma^2,\\
\Gamma &= (\Gamma_c\beta^2\sigma_c^2 + \Gamma_m\sigma_m^2)/\sigma^2,\\
r &= (r_c\beta^2\sigma_c^2 + r_m\sigma_m^2)/\sigma^2,\\
c &= (c_c\beta^2\sigma_c^2 + c_m\sigma_m^2)/\sigma^2,
\end{aligned} \tag{35}$$

where $\tau = \sigma_c\sigma_m \mathrm{Cor}(c_{ij}, m_{ij})$. We have assumed in (35) that the correlations between other pairs of elements coming from each random matrix in (33) are null for simplicity (for instance, $\mathrm{Cor}(c_{ij}, m_{ji}) = 0$); the corresponding generalization would follow analogously. Note that we have chosen a different scaling in N from that in Baron *et al* [6]. This produces a deformation of the ellipse on which the bulk of the eigenvalues is uniformly distributed, as shown in Fig. 5 of the main text. The location of the outlier eigenvalue is nevertheless correctly estimated after performing the corresponding rescaling in equation (32), following an analogous reasoning as the one in section 1.1 for the circular law and the low-rank perturbation theorem. After correcting for the different

diagonal terms in our model, this results in the following expression for the epidemic threshold for systems displaying the correlations in Eq. (34)

$$s(J) = \beta\mu_c(N-1) + \beta - \gamma + \frac{N}{2}\left(\beta\mu_c + \mu_m\right)\left(1 + \frac{\rho}{N\Gamma}\right)\left(\sqrt{1 + \frac{4\Gamma\sigma^2}{(\beta\mu_c + \mu_m)^2}} - 1\right). \tag{36}$$

See Fig. 5 in the main article for examples of this outlier eigenvalue and the effect of correlations in the stability of the system. Whenever both correlations Γ_c and Γ_m are zero, the last term in Eq. (36) vanishes and one recovers the original expression for the stability threshold Eq. (11). In the more general case, the sign of Γ determines whether the correlations facilitate or prevent the spread of the disease over the network (Fig. 15). We see from (36) that the epidemic threshold also depends on the correlation ρ between mobility flows going in opposite directions, as well the average migration rate and the joint variability of the connectivity network (Fig. 16).

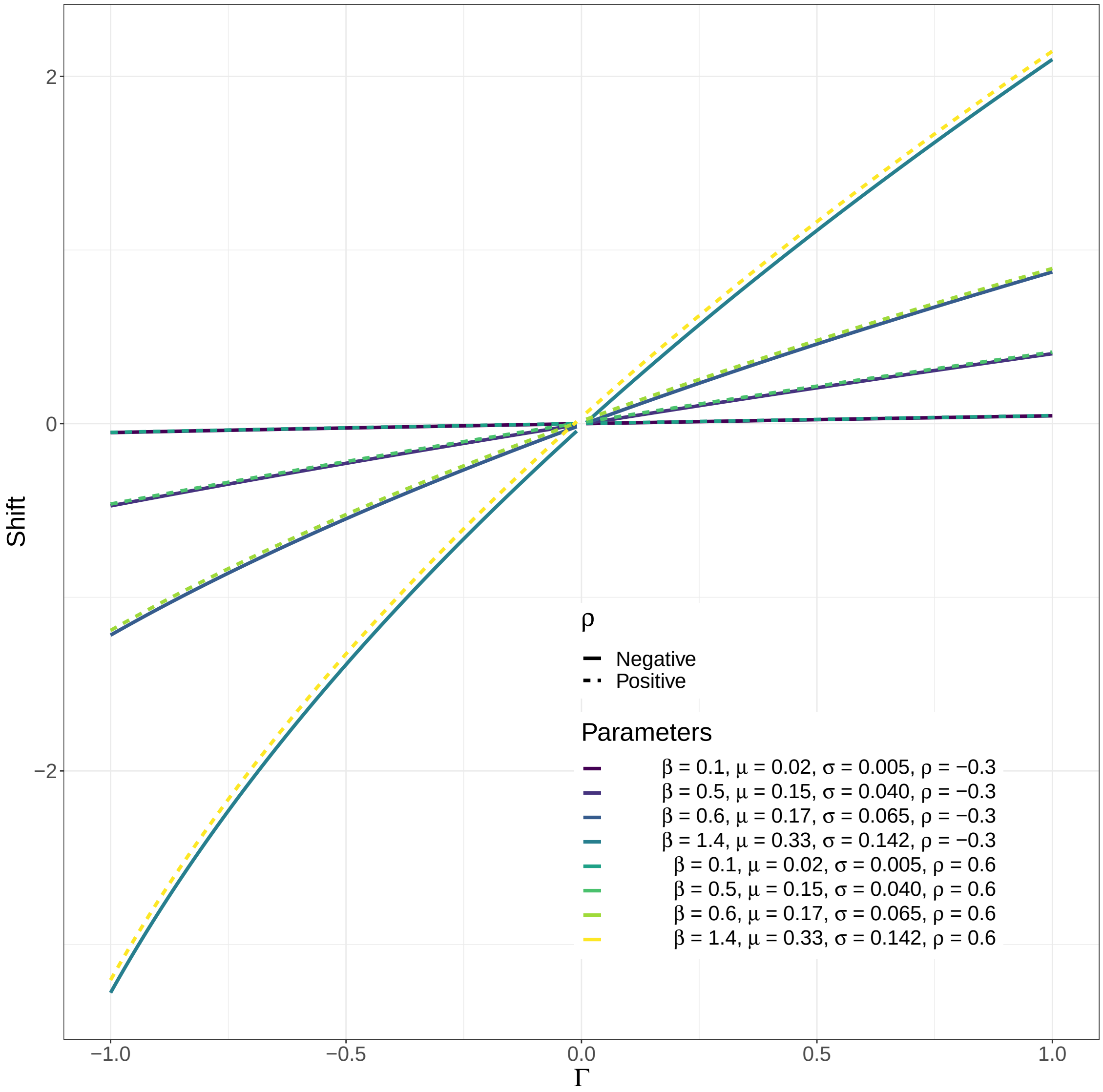

Figure 15: **Correlated mobility flows shift the epidemic threshold.** Size of the perturbation of the outlier eigenvalue (given by the last term in Eq. (36)) in terms of the joint correlation Γ for several choices of transmission rate β and joint mean and variance of the mobility network ($\mu = \beta\mu_c + \mu_m$, $\sigma^2 = \beta^2\sigma_w^2 + 2\beta\tau + \sigma_c^2$). Negative (positive) values for this shift mean that the epidemic threshold for the correlated system is smaller (larger) than that expected from the unperturbed expression Eq. (11). The relatively small change in stability caused by ρ can be seen from the fact that a change from $\rho = -0.3$ (solid lines) to $\rho = 0.6$ (dashed lines) produces little variation in the outlier eigenvalue. In all cases, the sign of Γ determines whether the correlated network will be more stable (positive shift in $s(J)$) or less stable (negative shift in $s(J)$).

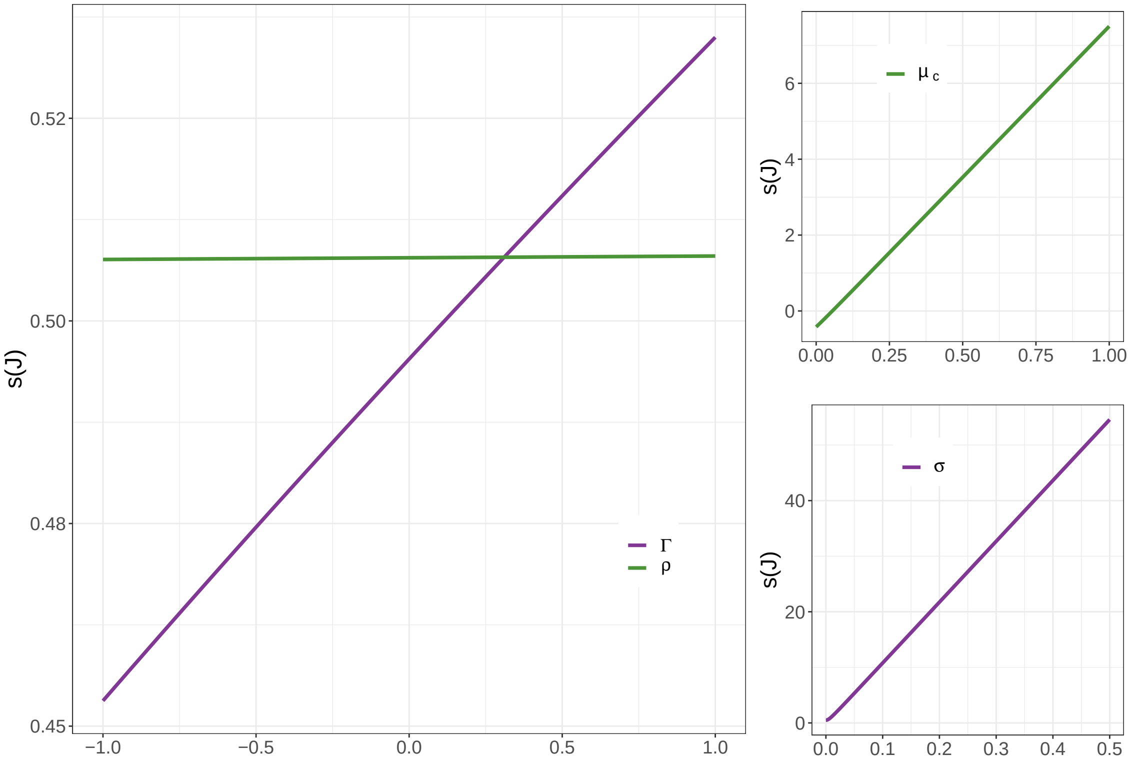

Figure 16: **Epidemic threshold $s(J)$ for systems with correlated connectivity networks.** Changes in Eq. (36) upon variations in several parameters of the system: the correlation between incoming and outgoing flows at the patches Γ, the correlation between flows going in opposite directions ρ, the average commuting flow μ_c and the joint standard deviation of the connectivity networks σ. Note the different y scales in the three graphs; parameters for the base system are $N = 200, \Gamma = 0.295, \rho = 0.44, \mu_c = 0.12, \sigma = 0.0012$. As observed for systems with only nontrivial ρ correlation between their connectivity flows, the effect of this parameter on the epidemic threshold vanishes asymptotically (Eq. (36)).

References

[1] Stefano Allesina, Jacopo Grilli, György Barabás, Si Tang, Johnatan Aljadeff, and Amos Maritan. Predicting the stability of large structured food webs. *Nature communications*, 6(1):1–6, 2015.

[2] Stefano Allesina and Si Tang. Stability criteria for complex ecosystems. *Nature*, 483(7388):205–208, 2012.

[3] Roy M Anderson and Robert M May. *Infectious diseases of humans: dynamics and control.* Oxford university press, 1992.

[4] J. Arino, J. R. Davis, D. Hartley, R. Jordan, J. M. Miller, P. van den Driessche, and James Watmough. A multi-species epidemic model with spatial dynamics. *Mathematical medicine and biology : a journal of the IMA*, 22(1):129–142, 2005.

[5] Joseph W Baron and Tobias Galla. Dispersal-induced instability in complex ecosystems. *Nature communications*, 11(1):1–9, 2020.

[6] Joseph W Baron, Thomas Jun Jewell, Christopher Ryder, and Tobias Galla. Eigenvalues of random matrices with generalized correlations: A path integral approach. *Physical Review Letters*, 128(12):120601, 2022.

[7] Rajendra Bhatia and Chandler Davis. A better bound on the variance. *The american mathematical monthly*, 107(4):353–357, 2000.

[8] Fred Brauer, PD Driessche, and Jianhong Wu. Lecture notes in mathematical epidemiology. *Berlin, Germany Springer*, 75(1):3–22, 2008.

[9] O. Diekmann, J. A. P. Heesterbeek, and J. A. J. Metz. On the definition and the computation of the basic reproduction ratio r0 in models for infectious diseases in heterogeneous populations. *Journal of Mathematical Biology*, 28(4):365–382, Jun 1990.

[10] O. Diekmann, J. A.P. Heesterbeek, and M. G. Roberts. The construction of next-generation matrices for compartmental epidemic models. *Journal of the Royal Society Interface*, 7:873–885, 6 2010.

[11] Peng Du and Michael Y Li. Impact of network connectivity on the synchronization and global dynamics of coupled systems of differential equations. *Physica D: Nonlinear Phenomena*, 286:32–42, 2014.

[12] Peter J. Forrester. *Log-Gases and Random Matrices (LMS-34)*. London Mathematical Society Monographs, London, 2010.

[13] Ying He, Fei Richard Yu, Nan Zhao, Hongxi Yin, Haipeng Yao, and Robert C Qiu. Big data analytics in mobile cellular networks. *IEEE access*, 4:1985–1996, 2016.

[14] M.J. Keeling and P. Rohani. *Modelling Infectious Diseases.* Princeton University Press, 2008.

[15] William Ogilvy Kermack and Anderson G McKendrick. A contribution to the mathematical theory of epidemics. *Proceedings of the royal society of london. Series A, Containing papers of a mathematical and physical character*, 115(772):700–721, 1927.

[16] Diana Knipl. A new approach for designing disease intervention strategies in metapopulation models. *Journal of biological dynamics*, 10:71–94, 2016.

[17] Alun L. Lloyd and Robert M. May. Spatial heterogeneity in epidemic models. *Journal of Theoretical Biology*, 179(1):1–11, 1996.

[18] Robert M May and Roy M Anderson. Spatial heterogeneity and the design of immunization programs. *Mathematical Biosciences*, 72(1):83–111, 1984.

[19] Carl D Meyer. *Matrix analysis and applied linear algebra*, volume 71. Siam, 2000.

[20] Hoi H Nguyen and Sean O'Rourke. The elliptic law. *International Mathematics Research Notices*, 2015(17):7620–7689, 2015.

[21] Sean O'Rourke and David Renfrew. Low rank perturbations of large elliptic random matrices. *Electronic Journal of Probability*, 19:1–65, 2014.

[22] Lisa Sattenspiel and Klaus Dietz. A structured epidemic model incorporating geographic mobility among regions. *Mathematical biosciences*, 128(1-2):71–91, 1995.

[23] Lisa Sattenspiel and Alun Lloyd. *The geographic spread of infectious diseases: models and applications*, volume 5. Princeton University Press, 2009.

[24] Peter Shaffery, Bret D Elderd, and Vanja Dukic. A note on species richness and the variance of epidemic severity. *Journal of Mathematical Biology*, 80(7):2055–2074, 2020.

[25] Ignacio Taguas, José A Capitán, and Juan C Nuño. Dropping mortality by increasing connectivity in plant epidemics. *Physical Review E*, 105(6):064301, 2022.

[26] Terence Tao. Outliers in the spectrum of iid matrices with bounded rank perturbations. *Probability Theory and Related Fields*, 155(1):231–263, 2013.

[27] Terence Tao, Van Vu, and Manjunath Krishnapur. Random matrices: Universality of esds and the circular law. *The Annals of Probability*, 38(5):2023–2065, 2010.

[28] P. van den Driessche and James Watmough. Reproduction numbers and sub-threshold endemic equilibria for compartmental models of disease transmission. *Mathematical Biosciences*, 180(1):29–48, 2002.

[29] Richard S. Varga. *Geršgorin and His Circles.* Springer Berlin, Heidelberg, Heidelberg, 2004.

[30] Yan Wan, Sandip Roy, and Ali Saberi. Designing spatially heterogeneous strategies for control of virus spread. *IET Systems Biology*, 2(4):184–201, 2008.
